# Long-run electricity consumption in computing: Exponential growth followed by stabilization due to efficiency gains

**DOI:** 10.1016/j.isci.2026.114876

**Published:** 2026-02-03

**Authors:** Ricardo Pinto, Paul E. Brockway, Tiago Domingos, Tânia Sousa

**Affiliations:** 1 MARETEC—Marine, Environment and Technology Center, LARSyS - Laboratory for Robotics and Engineering Systems, Instituto Superior Técnico, Universidade de Lisboa, 1049-001 Lisboa, Portugal; 2 Sustainability Research Institute, School of Earth, Environment and Sustainability, University of Leeds, Leeds LS2 9JT, UK

**Keywords:** electricity, information and communication technologies, energy management

## Abstract

Published projections suggest that information and communication technologies could account for up to 20% of global electricity use by 2030, yet these estimates are often based on short (<5 years) historical periods. Here, we present the first global long-run (1975–2022) analysis that jointly estimates electricity consumption, processed information, and efficiency. We find that these increased by 4, 11, and 7 orders of magnitude, respectively. However, after an initial exponential growth, the share of computing devices in world electricity consumption peaked at 2.5% in 2013, then decreased and stabilized at 1.8% since 2018. The stabilisation was due to the massive increases in information processing being offset by efficiency gains associated with the growing amount of computation in large datacenters and the shift from desktop computers to laptops and, more recently, to smartphones. These results indicate that concerns about the future electricity demand of computing may be overstated.

## Highlights

• First global 1975–2022 joint analysis of electricity use, information, and efficiency
• Electricity, information, and efficiency rose by 4, 11, and 7 orders of magnitude
• Energy efficiency gains enabled past increases in information processing
• Computing devices consumed less than 2% of the world's electricity in 2022

## Graphical abstract

**Figure:**
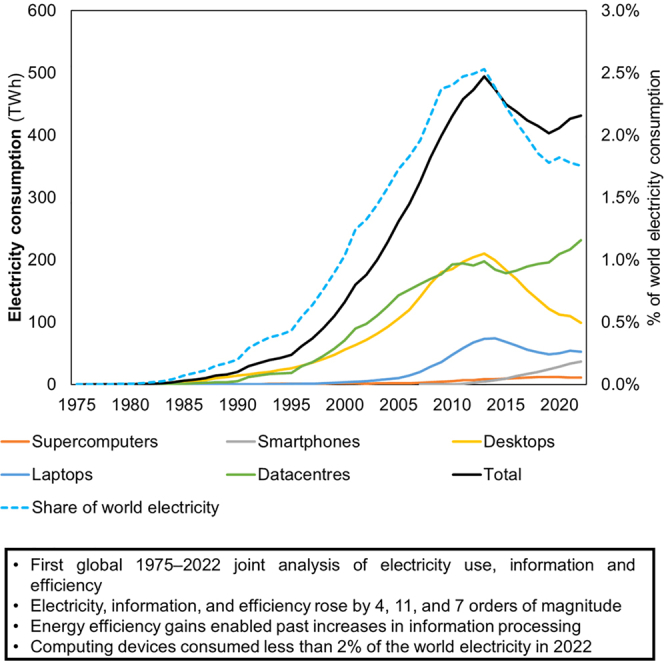

## Introduction

Studies about the ongoing energy transition have been primarily focused on the supply side^1^^,^^2^^,^^3^^,^^4^ or in large consumption sectors,^5^ such as housing and transport.^6^ Nonetheless, the energy transition will affect other sectors, for example, information and communication technology^7^ (ICT), which could significantly grow its electricity consumption. Consequently, increasing concern has emerged in recent years over the amount of electricity consumed by ICT, with several studies estimating its energy usage^8^^,^^9^^,^^10^^,^^11^^,^^12^^,^^13^^,^^14^^,^^15^^,^^16^ to represent between 4% and 7% of the world’s electricity in 2020.^12^^,^^14^ One of the main reasons for concern about ICT is its projected increased future energy demand.^17^ Future scenarios for ICT electricity consumption are highly dependent on the assumptions made about efficiency and ICT demands,^18^^,^^19^ with some estimates projecting that in 2030, more than 20% of the world’s electricity will be used in this sector.^16^

ICT is a broad category that can be divided into two main components: communication and computation. Communication refers to the transfer of bits of information from sources to destinations, while computation is any transformation done to bits of information.^20^ In this article, the scope is exclusively computation, specifically desktops, laptops, datacenters, smartphones, and supercomputers, which account for approximately 50% of the total electricity consumption in the ICT sector.^8^^,^^9^^,^^10^ The remaining ICT energy usage comes from the communication sector, with communication networks and modems being the primary contributors to electricity consumption.^8^^,^^9^^,^^10^

**Figure 1:**
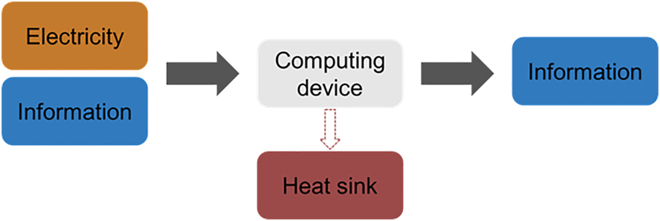
Energy and information flows in a computing device Schematic representation of the energy and information flow in a computing device, adapted from figure 8 in Zhirnov et al.^21^

When discussing any device’s current and future electricity consumption, it is important to consider its efficiency and the potential for improvements. Usually, the energy efficiency of any device is calculated by dividing the useful energy output by the energy input; however, in computing devices, there is no useful energy output (see Figure 1), only information.

When studying the energy efficiency of computing devices, most studies present results in the form of computations/instructions/operations per unit of energy. This metric represents relative efficiency but does not offer insights into absolute potential energy savings. Therefore, here we measure energy efficiency by comparing the electricity consumed per computation with the theoretical minimum energy required to do any irreversible change in information stored in a computer (switching a bit), known as the Landauer limit.^22^^,^^23^^,^^24^^,^^25^ This limit is independent of technology, so it provides a universal baseline against which all present and future computing technologies can be compared.

The first key limitation in previous historical studies of electricity consumption of computing devices is that they focus only on small timescales, i.e., a single year,^9^^,^^14^ a comparison between two years^10^ and a 5-year period.^8^ Those that present longer time periods only consider one type of computing device, e.g., datacenters^11^ (and only for an 8-year period) or only use historical data for the first years of the study,^12^^,^^16^ 2010 to 2013, with the remaining (later) years extrapolated.

The second key limitation is that studies that look at the electricity consumption of computers^8^^,^^9^^,^^10^^,^^11^^,^^12^^,^^13^^,^^14^^,^^15^^,^^16^ do not estimate efficiency, while conversely the studies that estimate efficiency^23^^,^^25^^,^^26^ do not include global electricity consumption calculations, focusing only on the device level consumption. An example is Andrae,^12^^,^^16^ who assumes efficiency will decrease the annual consumption per device by a certain percentage per year, depending on the scenario in question (a larger decrease in the best-case scenario when compared to the worst-case scenario), but never explicitly estimates efficiency.

In the previous paragraphs, we highlighted the uncertainties and gaps in existing studies of historical and future electricity consumption of computing devices and efficiency trends. These uncertainties need to be addressed so that future energy forecasts are based on more robust data and produce forecasts that can better inform policymakers. Such historical data are vital because one of the main questions of the ongoing energy transition is how much electricity we will need, with computing devices being one of the areas of expected growth for electricity demand. Better estimates of future electricity needs are essential for planning the infrastructure needed to produce and distribute it and to design effective policies that ensure investments are cost-effective and benefits are distributed.^27^ To address these gaps, we develop a comprehensive global study using a consistent and transparent methodology to examine the long-run evolution of electricity use and efficiency in computing. This study produces a global dataset encompassing electricity consumption, information processing, and energy efficiency metrics for various computing devices— desktops, laptops, supercomputers, servers, and smartphones—covering the 47-year period 1975 to 2022.

### Information, energy, and efficiency of computing devices

The initial data necessary to construct the dataset is the number of units of each type of computing device. We collected data about the installed base (stock) and, when available, the annual installation of new computing devices for each type. Additionally, we collected information on the number of hours various types of computing devices were used per year.

A crucial first step in calculating the information processed by a computing device is the assessment of its performance. Although computer performance is a complex topic, this article focuses on full-load computing capacity, in line with other historical studies.^26^^,^^28^ Various performance metrics and benchmarks are available; we use million instructions per second (MIPS), a widely used metric,^26^^,^^28^ and convert to this unit as needed, primarily from floating-point operations per second (FLOPS).

Regarding electricity consumption, we collected power data for computing devices for which we already had performance values. The electricity consumption of computing devices is a complex subject, as rated power and actual power consumption can differ significantly. Depending on the assumptions made, the estimates of annual electricity consumption of computing devices can vary significantly.^29^ We assumed that the power consumption of personal computers (PCs), which include desktops and laptops, is 50% of the rated power based on previous studies.^30^^,^^31^

Finally, using data on the number of computing devices, their performance, power consumption, and hours of use, we calculated annual electricity consumption and information processed each year. To obtain a yearly relative efficiency measurement in Joules per bit, we divided the annual electricity consumption by the annual amount of information processed. We then assessed absolute energy efficiency by dividing the theoretical limit, Landauer limit, by the relative efficiency, as shown in Equation 1, where k is Boltzmann’s constant, and T is the absolute environmental temperature.

(Equation 1) $$ƞ=\frac{k\;T\;\ln\;2}{relative\;efficiency\;}$$

## Results

### Number of devices and information computed

**Figure 2:**
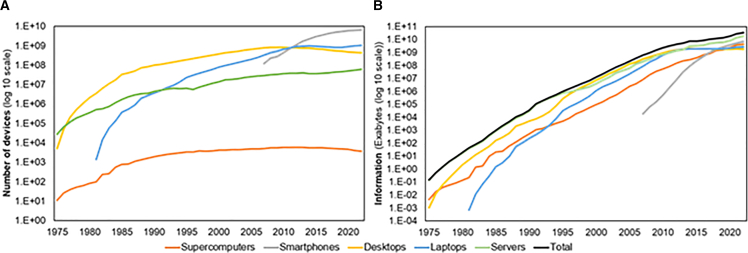
Number of devices and information computed (A) World historical number of computing devices, 1975 to 2022. (B) Global evolution of computed information by device type. Both graphs are on a base-10 logarithmic scale. See also Tables S11, S12, S21, and S22.

Historically, the number of computing devices has increased from roughly thirty thousand in 1975 to close to 8 billion in 2022 (Figure 2). Smartphones are the most widely owned computing devices, and there is an overall clear trend toward more mobile devices. This shift began with a move from desktops to laptops and has more recently extended to smartphones. Technological progress enabled the increase of laptops’ batteries^32^ that, together with a user desire for a more mobile computing device, explains the growth seen in the share of laptops (see Figure S9 of SI) at the expense of desktops. The next shift, the increase in the number of smartphones, is also related to technological progress, which enabled smartphones to reach performance capabilities close to laptops.^33^ Smartphone performance improvement enabled the use of applications that were previously only available on laptops. The shift in behavior is also observed in the transition from residential laptop use to smartphone use, as reflected in the usage hours (see Tables S20 and S27).

From 1975 to 2022, the annual global computed information increased by more than 11 orders of magnitude (Figure 2), a compound annual growth rate (CAGR) of 74.52%. Today, servers are the devices that handle and store the most information (see Figure S10 of SI for the evolution of the share of each type of device); this trend is expected to continue, as data traffic is projected to rise in the coming years.^18^ Comparing our results with the Hilbert and López^34^ study that quantified computed information only for the benchmark years 1986, 1993, 2000, and 2007, we find that our estimates align in order of magnitude for their first year (1986) but are one order of magnitude higher for the remaining three years (1993, 2000, 2007) of that study^34^ (see Tables S1–S4 for a comparison of our results with Hilbert and López^34^). This discrepancy arises from differences in our estimates of computed information in two types of computing devices: servers and personal computers (PCs), which include desktops and laptops.

Computed information, for PCs and servers, is estimated by multiplying three parts: the stock (in units), the hours of use (per year), and the average performance (in MIPS) (see more detail in the section titled Data/Methods S1 of the supplemental information). First, regarding stocks, 1993 is the only year showing significant discrepancies and only in server estimates (PC stocks are always very similar); our value is 60% of that reported by Hilbert and López^34^ (see Table S1 of the supplemental information).

Second, for residential PC usage hours, we use the same values as Hilbert and López.^34^ However, unlike them, we distinguish between residential and office PCs (see the section titled Data/Methods S1 of the supplemental information), as they assume all PCs are residential. We assume office PCs are used 8 h per day, 5 days a week. This distinction explains most of the differences in the estimated computed information for 1993 and 2000. Regarding servers' hours of use, we assume that servers work 24 h per day every day, based on Masanet et al.,^11^ while Hilbert and Lopéz^34^ assume only 8 h per day, which explains almost completely the differences in computed information for 1993 and 2000.

Third, the difference between our average performance estimates and those of Hilbert and López^34^ arises from the differing methods used. Our estimates of average performance were based on previous historical studies^26^^,^^28^^,^^35^ that utilized various benchmark values to convert computer performance into MIPS. If computer A has a performance value measured in both MIPS and a specific benchmark, this relationship can be used to determine the MIPS performance of any other computer evaluated with the same benchmark. Hilbert and Lopéz^34^ used only one factor to convert to MIPS; in addition, for 2007, they used linear extrapolation since their performance dataset ended in 2006.

For 2007, the difference in computed information by PCs and servers is explained by a combination of hours of use and average performance, with our values being respectively 2-fold and 5-fold the ones obtained by Hilbert and Lopéz.^34^

### Electricity consumption of computing devices

**Figure 3:**
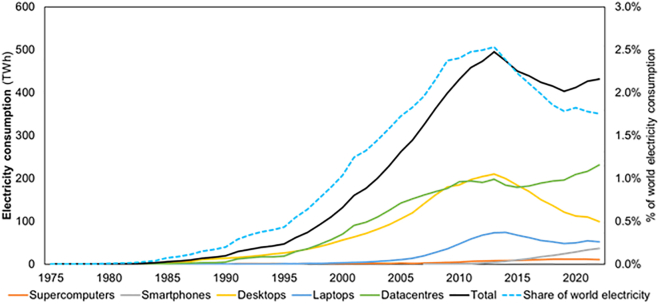
World annual electricity consumption of computing devices Global electricity consumption (TWh/yr) for each type of device, 1975 to 2022, datacenters include the electricity consumption of servers, internal networks, and infrastructure (left axis); computing devices' share of world electricity consumption, 1975 to 2022 (right axis). World electricity consumption data were retrieved from Pinto et al.^36^ prior to 2017 and from IEA^37^ afterward. See also Tables S13, S24, and S32.

The electricity consumption of computing devices increased by 4 orders of magnitude during the studied period (Figure 3), a CAGR of 24.40%, with PCs and datacenters, which include servers, cooling, and internal networks, being responsible for most of the electricity consumption. Initially, as desktop numbers rose, so did their share in electricity consumption (see Figures S9 and S11 of the supplemental information), to a peak of 75%; after 1990, their share declined until 2000, because of an increase in the share of datacenters, which became the predominant device in electricity consumption. Between 2005 and 2015, laptops’ share rose because of increases in the number and hours of use of residential laptops, at the expense of a decline in the share of datacenters, while desktops' share stayed stable. After 2016, datacenters became the predominant type of computing device again, with a growing share until 2022. This increase in the share of datacenters coincides with their increase in share in computed information and was caused by the introduction of cloud computing. In the last year of our study, smartphones and supercomputers together account for just over 10% of computing electricity use, which is the maximum they have represented over the entire period. Comparing our results for the sum of electricity consumption of desktops, laptops, datacenters, and smartphones, we can see that it differs by less than 10% from the expected scenario of Andrae^12^ for the period 2010 to 2015 (see Table 1) (the expected and best scenario differ in their assumption about energy efficiency).

**Table 1:** Electricity consumption of computing devices

	Electricity consumption (TWh)		
	Andrae^12^		Our estimate
	Best	Expected	
2010	336	441	426
2011	340	446	451
2012	369	477	466
2013	359	467	486
2014	356	480	465
2015	354	496	441
2016	352	513	428
2017	350	531	413
2018	349	551	403
2019	350	573	391
2020	352	604	400
2021	343	624	415

Comparison between our results and Andrae^12^ results for the total electricity consumption of computing devices.

### Energy efficiency of computing devices

**Figure 4:**
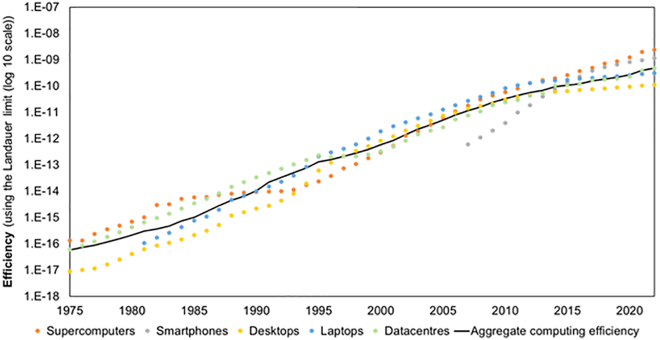
Energy efficiency of computing devices World average energy efficiency for each type of device, 1975 to 2022. Efficiency is calculated by comparing with the Landauer limit. The graph is on a base-10 logarithmic scale. See also Table S14.

Our results show a continuous increase in efficiency over time, though at varying rates across different types of devices (Figure 4). Overall, aggregate efficiency increased by 7 orders of magnitude between 1975 and 2022 (Figure 4), a CAGR of 40.30%. The energy efficiency of information processing has grown throughout the whole period, but this growth is initially lower in 1975–1983 (CAGR = 29.57%), higher in 1983–2005 (CAGR = 52.54%), and then exhibits a decreasing CAGR, reaching 24.23% in 2013–2022. This overall growth in efficiency is the result of a changing mix of computing devices and of the evolution of their respective efficiencies. In the period 1983–2005, efficiency growth was dominated by the increases in desktops and laptops’ efficiency, which processed the majority of information. These two types of computing devices benefited from the introduction of the metal-oxide-semiconductor field-effect transistor (MOSFET) during the 1980s,^38^ and the miniaturization of these transistors following Dennard scaling.^39^ This enabled clock frequency to go up, which enabled performance to increase while reducing power per transistor^40^ (at the chip level, power consumption was constant because the number of transistors correspondingly doubled). Between 2003 and 2005, clock frequency stagnated, and the efficiency of desktops’ and laptops started to grow at a slower pace. From 2013 onwards, the efficiency in laptops and desktops stagnated, but the efficiency in smartphones increased rapidly. In this period, after 2013, smartphones represented a significant portion of information processed, and they pushed efficiency up.

Focusing on PCs, there was a 6-orders-of-magnitude increase in efficiency from 1975 to 2009, consistent with the findings of Koomey and colleagues,^26^ who found a similar increase over the same period in computations per kWh for a set of devices composed mostly of PCs. During the entire period, our results show PC efficiency doubling approximately every 2 years. However, since 2000, the pace of improvement has slowed, with efficiency doubling roughly every 3 years – a trend also pointed out by Koomey.^41^

A comparison with Prieto et al.^25^ reveals small differences in the doubling time of supercomputer efficiency. Their results show efficiency doubling every 2.29 years, between 2008 and 2023, while our results show efficiency doubling every 2.23 years, between 2008 and 2022.

## Discussion

### Electricity consumption per type of computing device

**Figure 5:**
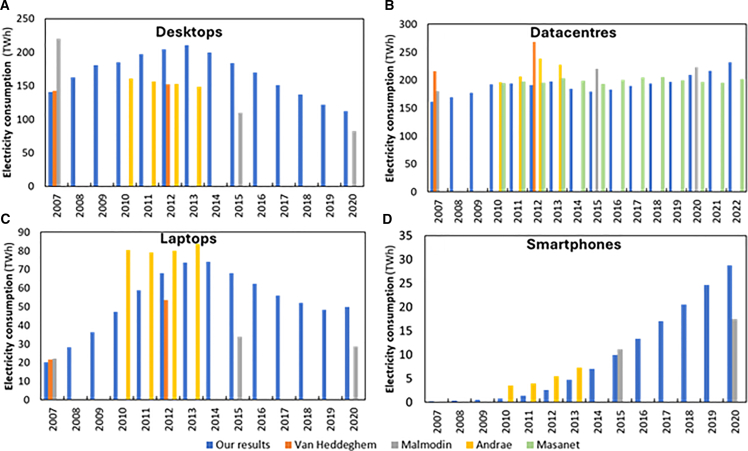
Annual electricity consumption per type of computing device (A) Electricity consumption of desktops. (B) Electricity consumption of datacenters. (C) Electricity consumption of laptops. (D) Electricity consumption of smartphones. In all plots, there is a comparison of the annual electricity consumption of each computing device with the literature.^8^^,^^10^^,^^11^^,^^12^^,^^14^^,^^42^ See also Table S15.

As shown in Figure 5, our results for the electricity consumption of each type of device are mostly in line with the literature. However, some discrepancies need to be further explained. Regarding desktops (Figure 5A), our results reach a maximum because of increases in the number of desktops (yellow line in Figure 2A) and in the hours of use of residential desktops (see Table S20), followed by the decline of both factors. The declining trend is in line with both Andrae^12^ and Malmodin.^10^^,^^14^^,^^42^ However, the absolute electricity consumption values differ slightly, as their annual consumption per device is lower (calculated from usage hours and power; a comparison with literature values is provided in Tables S5 and S6 of the supplemental information, and corresponding graphs (Figure S12) are also included there). Our results for power and hours of use are based on power data per desktop and internet use, respectively. Andrae^12^ assumes annual decreases in energy consumption without accounting for behavioral changes, whereas Malmodin^10^^,^^14^^,^^42^ reviews several studies reporting annual consumption per device but adopts a value near the lower end of those estimates. In addition, Malmodin et al.'s most recent studies^10^^,^^14^ use the same value, assuming no change occurred over the five-year period.

Concerning laptops (Figure 5C), we can see peaks in 2014 and 2021. The 2014 peak was caused by an increase in the hours of use of residential laptops and an increase in the number of laptops in use (blue line in Figure 2A). The 2021 peak is caused by an increase in the number of laptops in use when compared with 2019 (possibly due to increases in telework and homeschooling). Our results show that the annual electricity consumption of each laptop grows until 2013 due to the increase in hours of use for residential laptops and decreases after 2013 due to a decrease in hours of use. The differences in the total electricity consumption of laptops are explained by the discrepancies in the annual consumption of each individual laptop (see Figure S13 in the supplemental information), and they are especially visible when comparing with Malmodin and colleagues^10^^,^^14^^,^^42^ because they use a declining consumption of each individual laptop, while we use an increasing consumption until 2013, which only then starts to decrease. Malmodin et al.^14^ use a value for unit energy consumption in 2020 that is close to the lower end of the values they found in the literature (they use 25 kWh/year, but the literature they mention has values from 2 to 73 kWh/year), while our value, 56 kWh/year, is closer to the top end of the literature. The value assumed by Malmodin and colleagues^14^ assumes a power consumption for the whole laptop that is smaller than the power consumed by the average laptop processor, meaning no other system could consume power, and even the processor would have to work mostly below its capacity.

The electricity consumption of smartphones (Figure 5D) has increased since 2007 (the first year of our smartphone results). This is due to a continuous increase in the number of smartphones in use and of the annual electricity consumption of each individual smartphone (see Figure S14 in the supplemental information), which depends on the evolution of smartphones' battery capacity. The differences with Andrae^12^ are explained by the annual electricity consumption of each individual smartphone. Andrae’s values^12^ are more than double the average battery capacity, meaning that all smartphones would have to be fully charged more than two times per day. When comparing smartphones' electricity consumption with Malmodin et al.’s values,^10^^,^^14^ we obtain very similar results for 2015 but diverge in 2020. The divergence in 2020 is due to the fact that Malmodin and colleagues use the same value as in their previous study for the year 2015, meaning that they are disregarding the increased battery capacity of smartphones.^33^

For datacenters (Figure 5B), unlike other devices and time periods where all energy values were estimated by us, the energy values for 2010 to 2022 were based on the results of Masanet et al.,^11^ updated, after 2014, with the most recent values for the USA.^43^ This choice was made because Masanet et al.^11^ present more detailed data than what we have available (they have data about the number and power of subcategories of servers). Prior to 2010, we estimated the values based on power, number of devices, and hours of use, the same method that was used for the remaining types of devices, but at a higher aggregation level (see STAR Methods). Our results for the electricity consumption of datacenters closely align with those of Malmodin and colleagues.^10^^,^^14^^,^^42^ Similarly, our findings are consistent with those of Andrae^12^ for 2010 and 2011 but diverge significantly in subsequent years. This divergence stems from Andrae’s assumption that electricity consumption is correlated with network traffic when extrapolating data from 2010 onwards, an assumption that several authors have since questioned because electricity use increases are almost never proportional to growth in service demand.^44^^,^^45^^,^^46^^,^^47^ Our results indicate that electricity consumption in datacenters has been increasing more slowly than estimates from other authors^12^^,^^48^ whose values are based on the aforementioned correlation with network traffic or who are studying smaller geographical scales where trends could diverge from global trends.

The evolution of the electricity consumption of each type of computing device is also influenced by device replacement cycles because the stock of each device type is determined by annual additions and retirements. Shorter lifetimes accelerate the turnover of older, less efficient units, while longer lifetimes slow the diffusion of efficiency improvements into the global stock. For example, during this period, we observed that smartphones had an increase in their average lifetime from less than 2 years to more than 4 years.

### Sensitivity analysis

We conducted a sensitivity analysis where we analyzed several parameters (see Tables S7–S10 of the supplemental information for detailed information). For all types of computing devices, we increased/decreased the number of devices by 20% (see Figures S15, S16, S23, S27, and S31), we increased/decreased rated power/battery capacity by 20% (see Figures S19, S20, S25, S29, and S33), and we increased/decreased performance by 20% (see Figures S17, S18, S24, S28, and S32). For residential PCs (desktops and laptops) and smartphones, we also increased/decreased the usage time by 20%. For all PCs, we tested the assumption about 50% of rated power by assuming 25% and 37.5%, since the range from the literature was 25–50%.^30^^,^^31^ For PCs, we also tested device lifetime. All the results for these sensitivity tests are available in the supplemental information. These sensitivity analyses align with what Furberg et al.^47^ consider key aspects of assessments about direct energy use in the ICT sector.

We then performed a Monte Carlo simulation of electricity consumption, using the maximum and minimum values from all sensitivity tests for each device type and assuming a uniform distribution within that range. We summed the values of each type of device in each of the 10 000 simulations per year and then calculated the annual mean electricity consumption for each year (the raw data is available in the supplementary data file named Data S2).

**Figure 6:**
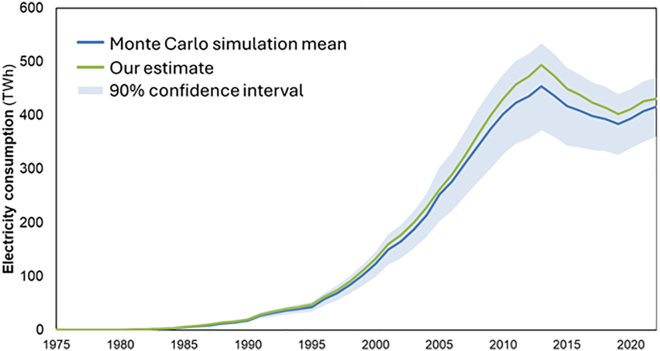
Monte Carlo simulation results for the electricity consumption of computing devices Comparison of the mean from the Monte Carlo simulation with our estimate. Shaded in light blue is the 90% confidence interval of the Monte Carlo simulation.

The Monte Carlo results are presented in Figure 6, where they are compared with our estimate. Our results for total electricity consumption are very similar to the mean of the Monte Carlo simulation and are clearly within the confidence interval. The small difference between the two lines shows that even with different assumptions/parameters, we obtain similar results.

### Future perspectives

Our results show that the electricity consumption of computing devices in the 2018–2022 period stabilized at 1.8% of the world's electricity consumption. During that period, the absolute value for the electricity consumption of computing devices has grown at a slow pace, less than 1% per year. In this period, processed information grew more than 28% per year, which meant that efficiency had to grow at the same rate to keep computing devices’ electricity demand constant. Assuming this trend continues into the future, the concerns about future energy needs of computing devices may be overstated, especially in scenarios where more than 10% of the world’s electricity is used in computing devices. Notwithstanding, an increase in electricity demand in any sector hampers the push for decarbonization as part of a global climate change mitigation effort.

However, sustained efficiency gains do not guarantee a continued stabilization of electricity demand. New services and applications can increase total computational activity, potentially leading to rebound effects where improved efficiency enables greater use rather than reducing energy needs. This dynamic could be currently being seen in the case of artificial intelligence (AI), where demand growth may be outpacing efficiency improvements. These findings suggest that long-term outcomes for ICT electricity demand will depend not only on efficiency progress but also on the rate at which new computational services expand. Monitoring total demand, alongside efficiency trends, is important for guiding future policy discussions regarding ICT sustainability.

Moreover, despite this apparent stabilization, future electricity demand of computing devices remains uncertain,^47^ with two components, AI and cryptocurrencies, being directly related to the concerns about future electricity demand, not having been evaluated here. Regarding AI, its electricity demand remains highly uncertain.^49^ While some estimates suggest global AI consumption ranges from 7 to 10 TWh per year,^50^ others place it at 40 TWh annually for the U.S. alone.^43^ However, recent developments in China (such as DeepSeek^51^^,^^52^) indicate that it is possible to significantly reduce the electricity consumption associated with AI model development and use. Furthermore, AI is a new/better service, and the experience with transitions to better services in other sectors is that they usually increase energy use.^53^ Cryptocurrencies have a significant electricity consumption of 100 TWh/year^54^ – nearly 25% of current computing electricity use. However, a change in the consensus mechanism, going from proof-of-work to proof-of-stake (which has already happened in the second biggest cryptocurrency), would drastically reduce their electricity consumption.^55^

### Insights

Our comprehensive global study on computing provides a long-term analysis of electricity consumption, efficiency, and information processing across various types of computing devices, using a consistent methodology. Our results indicate that electricity consumption for computing has grown at a slower rate than overall electricity demand, despite the continued increase in processed information. Since 2018, it has accounted for approximately 1.8% of global electricity use, after peaking at 2.5% in 2013. This slow increase was possible due to gains in efficiency, which more than doubled between 2018 and 2022, keeping pace with the growth in information processed. Efficiency improvements were mainly related to the shift to smartphones and the rise in large (hyperscale) datacenters. Our results suggest that current concerns about the future electricity demand of computing may be overstated.

As mentioned in the future perspectives section, there is a high uncertainty regarding current and future AI energy demand; these uncertainties can lead to a scenario where future electricity demand increases more rapidly. A good starting point to try to reduce these uncertainties, and a future work proposal, is to build future scenarios for datacenters’ electricity demand. These should include the latest data about AI datacenters already in operation and future additions planned. These scenarios should also take into account policy changes and electricity grid constraints.

The ICT sector as a whole, and computing in particular, is a rapidly evolving field with many uncertainties. However, our results reveal that a steep increase in electricity consumption does not seem likely in the near future, because efficiency improvements from the switch to smartphones and the rise of large datacenters are keeping pace with the growth in processed information, and there is still ample room for efficiency improvements, as revealed by comparison with the (absolute) Landauer limit.

### Limitations of the study

Energy consumption in a processor has two different components: dynamic power and static power. Static power has been reduced since 2011 and is now significantly smaller than dynamic power.^56^ However, dynamic power itself can be divided into two components: the power to switch transistors and the power to charge wire capacitances. Empirical studies show that the relative importance of these two components varies widely across technologies, from more than 50% of dynamic power due to interconnects in older CPUs^57^ to about 14% in modern GPUs at 28 nm, increasing to roughly 22% when scaled to 7 nm.^58^ The Landauer limit represents the minimum value only for transistor switching. Currently, the minimum energy for charging wire capacitances is five orders of magnitude above the Landauer limit.^59^ In practical terms, this means that treating the Landauer limit as a direct proxy for full device energy results in an underestimation of roughly five orders of magnitude for present technologies. Incorporating technology-dependent limits, such as interconnect and leakage energy, will be relevant for short to medium-term projections, where device performance is constrained by current architectures.

## Resource availability

### Lead contact

Further information and requests for resources should be directed to and will be fulfilled by the lead contact, Ricardo Pinto (ricardo.c.pinto@tecnico.ulisboa.pt).

### Materials availability

This study did not generate new unique reagents.

### Data and code availability

The authors declare that all data supporting the findings of this study are available in the supplementary data files. This article does not report any original code. Any additional information required to reanalyze the data reported in this article is available from the lead contact upon request.

## Acknowledgments

Ricardo Pinto’s work was supported by 10.13039/501100001871Fundação para a Ciência e a Tecnologia through the individual research grant SFRH/BD/146923/2019 and by Project Blockchain.PT – Decentralize Portugal with Blockchain Agenda (Project no. 51), funded by the Portuguese Recovery and Resilience Program (PRR). Tânia Sousa’s and Tiago Domingos’s time was funded by the 10.13039/501100000780European Union under the Horizon Europe program, Grant Agreement No. 101137914. Paul E Brockway’s time was partly funded by the UK Research Council under EPSRC Fellowship Award EP/R024254/1 and Leverhulme Trust Research Project Grant RPG-2024-357. This work was also supported by FCT/MCTES (PIDDAC) through projects UIDB/50009/2025, UIDP/50009/2025, and LA/P/0083/2020. We would like to thank Arlindo Oliveira and Mário Figueiredo for their feedback on an early version of the article.

## Author contributions

R.P., P.B., T.D., and T.S. conceptualized the study. R.P, P.B., and T.S. developed the methodology used to produce the dataset. R.P. collected the data, analyzed the dataset, and wrote the original draft. R.P. and T.S. discussed the results. P.B., T.D., and T.S. contributed to the data analysis and to the editing of the article draft.

## Declaration of interests

The authors declare no competing interests.

## STAR★Methods

### Key resources table

**Table:** 

REAGENT or RESOURCE	SOURCE	IDENTIFIER
**Deposited data**		

World electricity consumption data after 2017	International energy agency (IEA)	https://www.iea.org/data-and-statistics/data-tools/energy-statistics-data-browser?country=WORLD&fuel=Energy%20consumption&indicator=BiofuelConsBySector
World electricity consumption data before 2017	Pinto et al.^36^	https://doi.org/10.1016/j.energy.2023.126775.

### Method details

We have collected and processed data from an array of different sources that enabled the creation of the dataset used as a basis for the results presented. We will describe below a general methodology. Further details regarding the methods can be found in the supplemental information with a detailed methodology for each computer category (supercomputers, servers, laptops, desktops and smartphones).

Our methodology has three objectives: estimating the information processed by computers, calculating their electricity consumption, and obtaining their energy efficiency. Since both information and energy data were required to calculate efficiency (which was calculated using Equation 1), we divide the methodology into two parts. First, we explain how we obtained the values for the information processed by computers, then we outline the steps to calculate their electricity consumption.

#### Information methods

Information processed by computers was estimated using the 5-step methodology summarized in Figure S1 of the supplemental information (see Figures S3, S5, and S7 for detail about each type of computing device).

Step 1 is the estimation of the stock of computers, also called installed base, for each year, using the computer lifetime (i.e., the average number of years a computer is in use), and the number of computers installed each year. The computer lifetime was based on literature and varied across different categories of computers. The number of computers installed per year was obtained from the literature and from specialized computer publications. The computer stock in a specific year was estimated by summing the new computers installed in that year to the stock of the previous year while subtracting the obsolete computers, computers that have exceeded their expected lifespan (i.e., the number of computers installed in year i-n, where n is the expected lifespan). Equation 2 summarizes this calculation.

(Equation 2) $${Computer\;stock}_{i}={Computer\;stock}_{i-1}+{new\;computers}_{i}-{obsolete\;computers}_{i}$$

Step 2 is the calculation of the average performance of the computers (the number of instructions executed per second) introduced in a specific year. We collected performance data for various computers, within each category. Some of these data points were taken from historical studies.^26^^,^^28^ We complemented these data with publicly available performance data from different Standard Performance Evaluation Corporation (SPEC) benchmarks, as well as performance data from the Geekbench 6 and LINPACK benchmarks. The values obtained from these benchmarks were then used to estimate performance values in millions of instructions per second (MIPS). We then calculated the average performance for each year and used interpolation for the years where data was unavailable.

Step 3 is the calculation of the weighted average performance (WAP) of the stock of computers for each year. This step is necessary because the weighted average computer performance for any given year is the average of the current installed base, meaning it considers all the computers in use in that year. We calculated WAP by multiplying the average performance for a specific year by the number of computers installed in that year and then repeating this process for the last x years, with x being the computer lifetime. Finally, we divided this value by the total computer stock. Equation 3 summarizes this calculation, n is the computer lifetime.

(Equation 3) $$Weightedaverageperformance{\left(WAP\right)}_{i}=\frac{\sum_{j-n+1}^{j}{average\;performance}_{j}\times {No.\;of\;new\;computers}_{j}}{{Computer\;stock}_{i}}$$

Step 4 is the calculation of the number of computations for each year. This was done by multiplying the WAP, the computer stock and the average time of computer use in each year, as shown in Equation 4. The average time of computer use varied depending on the type of computer considered and changed annually for some categories.

(Equation 4) $${No.\;of\;computations}_{i}={WAP}_{i}\times {Computer\;Stock}_{i}\times {average\;time\;of\;computer\;use}_{i}$$

Finally, step 5 is the conversion from computations to bits. This conversion was made using a conversion factor that varied over the years depending on the word length of computers. Essentially, we multiplied the result of Equation 4 by this conversion factor.

#### Energy methods

The electricity consumed by computers was estimated using the method summarised in Figure S2 of the supplemental information, which is divided into 4 steps (see Figures S4, S6, and S8 for detail about each type of computing device).

Step 1, which estimates the stock of computers for each year, is the same as step 1 in the information methodology.

Step 2 is the calculation of the average power consumption of computers for each year; this step is analogous to step 2 from the information method, with the difference being that we used power instead of performance.

Step 3 is the calculation of the weighted average power of the stock of computers, which was similar to step 3 of the information methodology, with the difference that we used power and not performance.

Finally, step 4 is the estimation of electricity consumption. We estimated the electricity consumed by computers in each year of our study by multiplying the weighted average power, the average hours of use, and the number of computers in use.

The calculation of electricity consumption was based on various assumptions regarding the share or type of power that should be used (rated power, peak power, active power). These assumptions depended on the availability of power data and type of computer being considered. For example, for PCs, we assumed 50% of rated power based on previous studies.^30^^,^^31^ The range of values on those studies was 25–50%, but we chose 50% in order to have the worst-case scenario in terms of electricity consumption.

### Quantification and statistical analysis

Data regarding the mean, weighted mean, median and standard deviation of power consumption for the various types of computing devices is available in Tables S19, S29, S31, S33, S34, S35, and S36 of the supplementary data file. Data regarding the mean and weighted mean performance for the various types of computing devices is available in Tables S17, S18, S26, and S30. Data regarding the mean and weighted mean word length/instruction set for the PCs and smartphones are available in Tables S23 and S28. Tables S16 and S25 show the useful life of supercomputers and smartphones, respectively. Tables S20 and S27 show the hours of use of personal computers and smartphones.

## Supplemental information

Document S1. Figures S1–S35, Tables S1–S10, and Data/Methods S1

Table S11. Number of devices, related to Figure 2A

Table S12. Information computed, related to Figure 2B

Table S13. Data about world's annual electricity consumption of computing devices, related to Figure 3

Table S14. Data about the energy efficiency of computing devices, related to Figure 4

Table S15. Data about annual electricity consumption per type of computing device, related to Figure 5

Table S16. Useful life of supercomputers by year of installation

Table S17. Supercomputers performance

Table S18. Personal computers performance

Table S19. Personal computers rated power consumption

Table S20. Personal computers hours of use per year

Table S21. Number of new PCs and stock of PCs

Table S22. Shares of new PCs and shares of total PCs

Table S23. Personal computers word length

Table S24. Electricity consumption of personal computers

Table S25. Smartphones useful life

Table S26. Smartphone performance

Table S27. Smartphones' hours of use per year

Table S28. Smartphones instruction set

Table S29. Smartphones battery capacity

Table S30. Servers' weighted average performance

Table S31. Servers' weighted average power draw

Table S32. Datacenters energy consumption

Table S33. Processor power consumption

Table S34. Personal computers rated power consumption raw data points

Table S35. Supercomputers rated power consumption raw data points

Table S36. Smartphones battery capacity raw data points

Data S2. Results from the Monte Carlo simulation
