# Supplementary material for: Long-run electricity consumption in computing: Exponential growth followed by stabilization due to efficiency gains
Source: iScience. 2026 Feb 3;29(3):114876. doi: 10.1016/j.isci.2026.114876 (PMC12955200; doi:10.1016/j.isci.2026.114876)
Supplement: Document S1. Figures S1–S35, Tables S1–S10, and Data/Methods S1 [file mmc1.pdf]

iScience, Volume *29*

Supplemental information

Long-run electricity consumption in computing: Exponential growth followed by stabilization due to efficiency gains

Ricardo Pinto, Paul E. Brockway, Tiago Domingos, and Tânia Sousa

Supplementary information

Supplementary figures

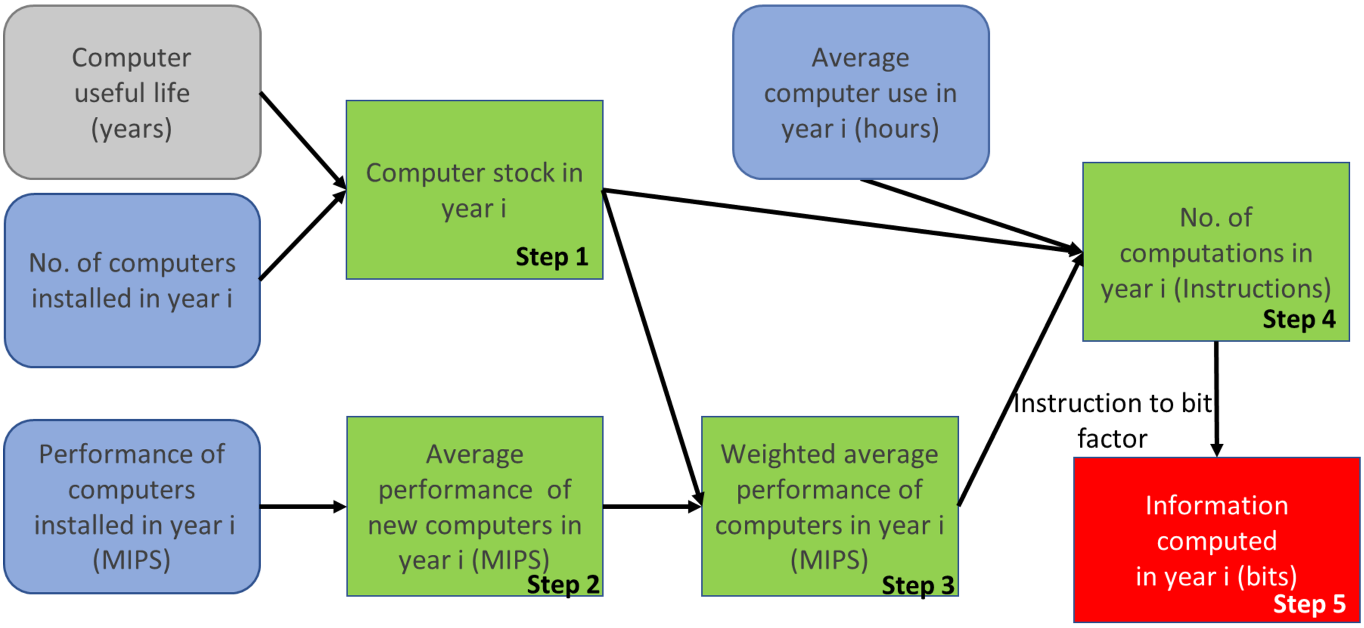

Figure S1 - Schematic representation of the methodology used to estimate information computed by computers. The background colours have the following meaning: Red – Objective; Green – Calculated value; Blue – Data collected; Grey – Assumptions based on the literature.

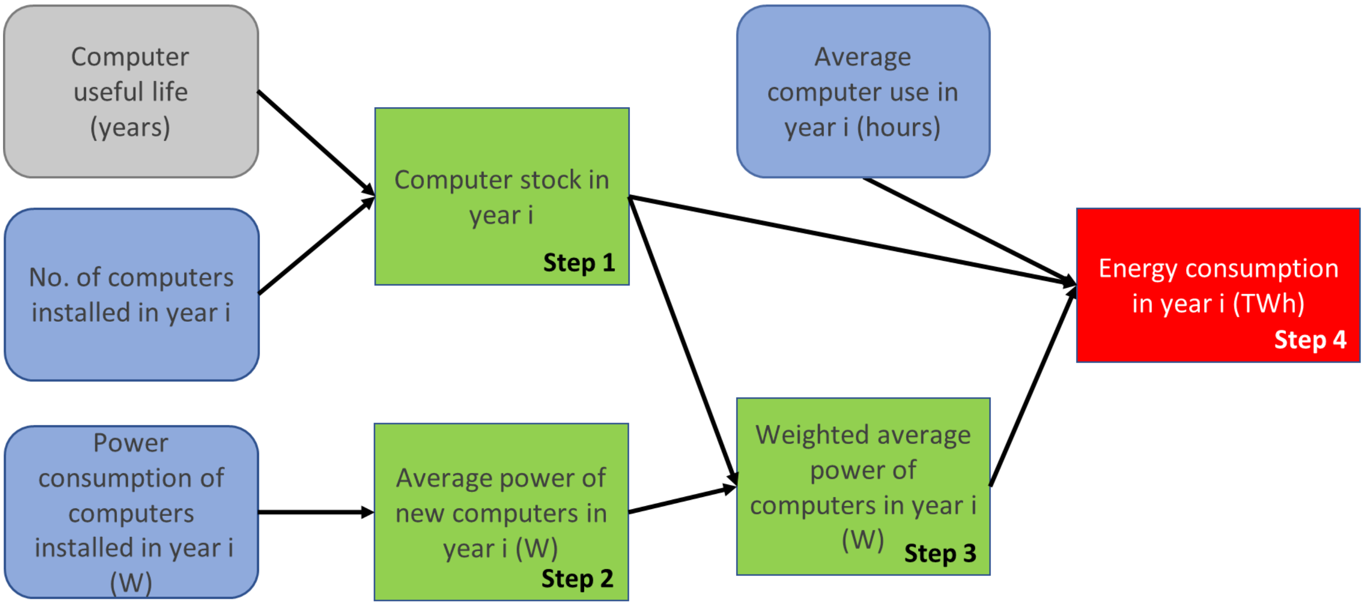

Figure S2 - Schematic representation of the methodology used to estimate electricity consumed by computers. The background colours have the following meaning: Red – Objective; Green – Calculated value; Blue – Collected data; Grey – Assumptions based on the literature.

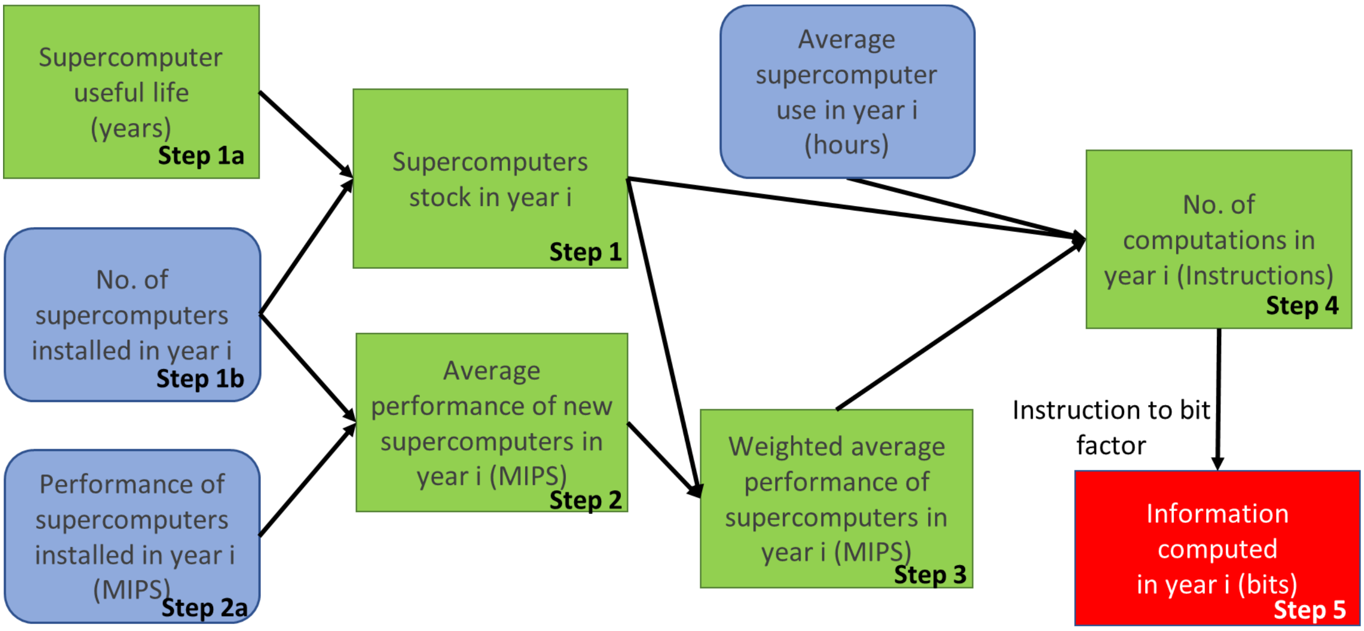

Figure S3 - Schematic representation of the methodology used to estimate information computed by supercomputers. The background colours have the following meaning: Red – Objective; Green – Calculated value; Blue – Collected data.

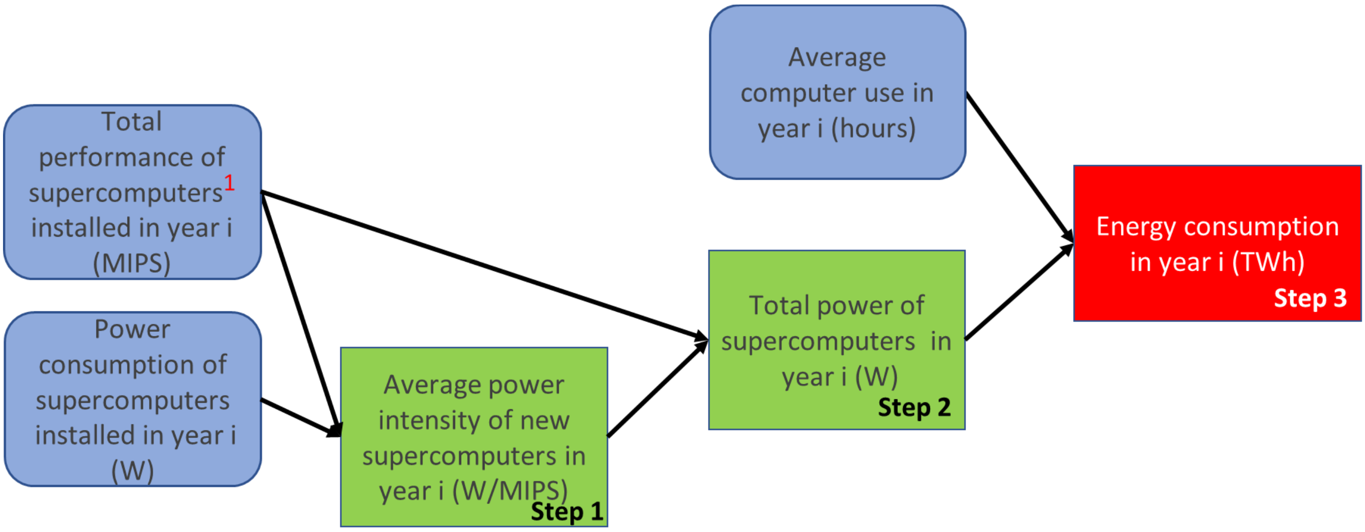

Figure S4- Schematic representation of the methodology used to estimate electricity consumed by supercomputers. The background colours have the following meaning: Red – Objective; Green – Calculated value; Blue – Collected data. 1- only computers with power data available.

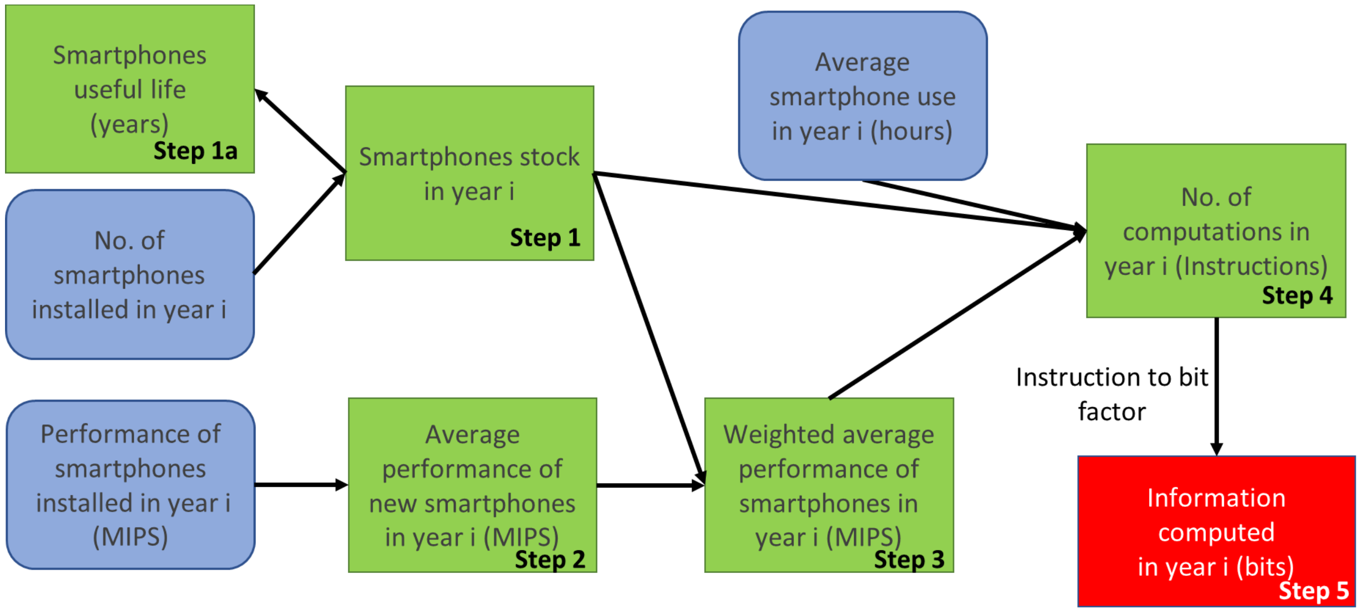

Figure S5 - Schematic representation of the methodology used to estimate information computed by smartphones. The background colours have the following meaning: Red – Objective; Green – Calculated value; Blue – Collected data.

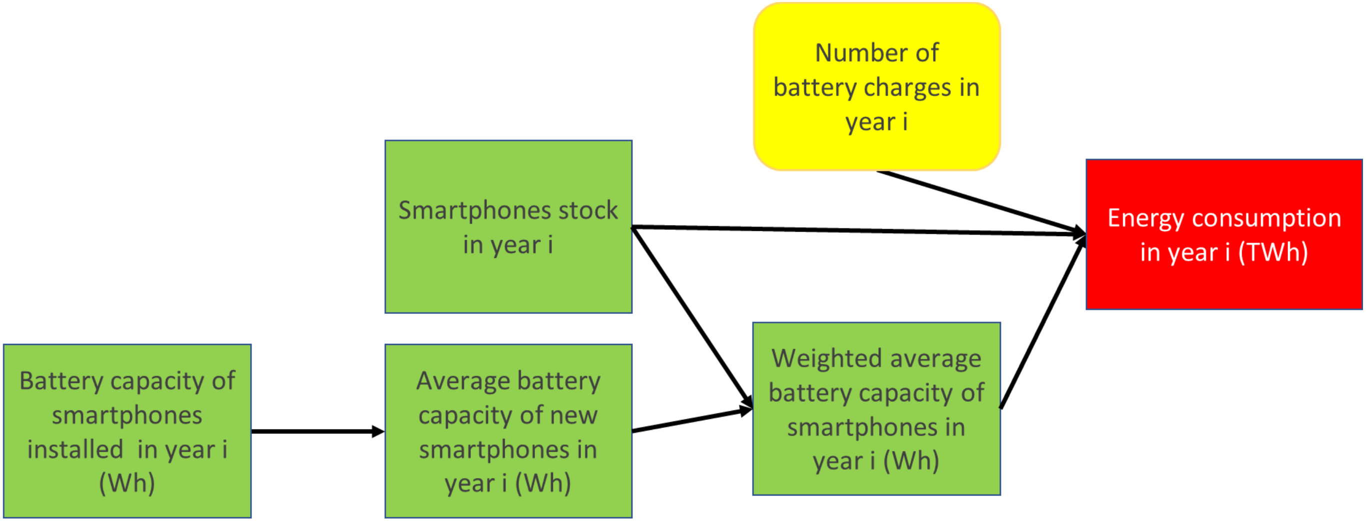

Figure S6 - Schematic representation of the methodology used to estimate electricity consumed by smartphones. The background colours have the following meaning: Red – Objective; Green – Calculated value; Yellow – Assumption

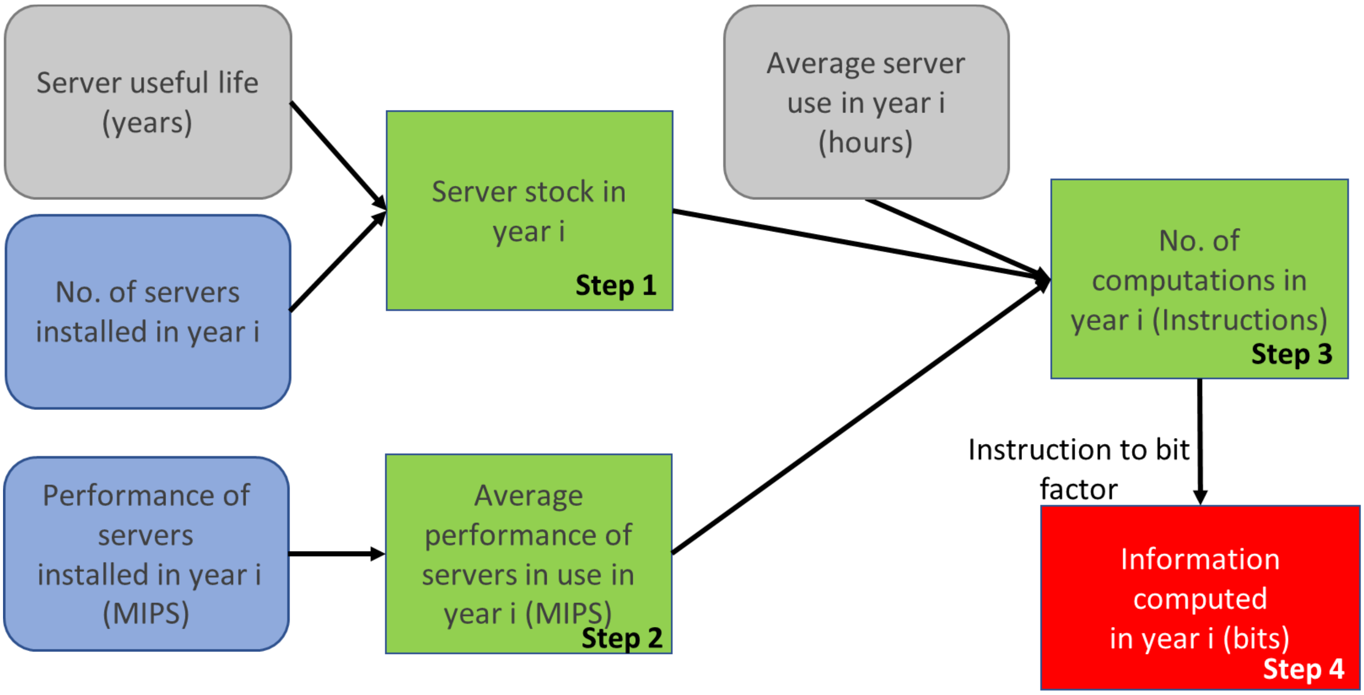

Figure S7 - Schematic representation of the methodology used to estimate information computed by servers. The background colours have the following meaning: Red – Objective; Green – Calculated value; Blue – Collected data; Grey – Assumptions based on the literature.

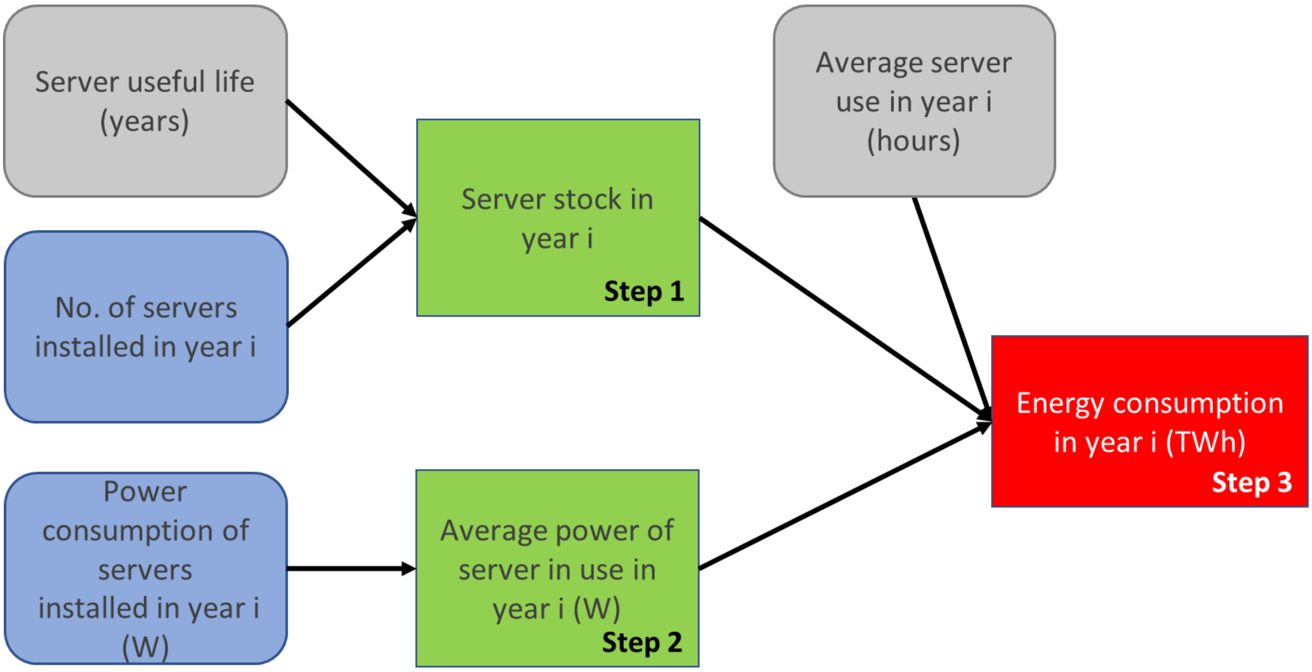

Figure S8 - Schematic representation of the methodology used to estimate energy consumed by servers. The background colours have the following meaning: Red – Objective; Green – Calculated value; Blue – Collected data; Grey – Assumptions based the on literature.

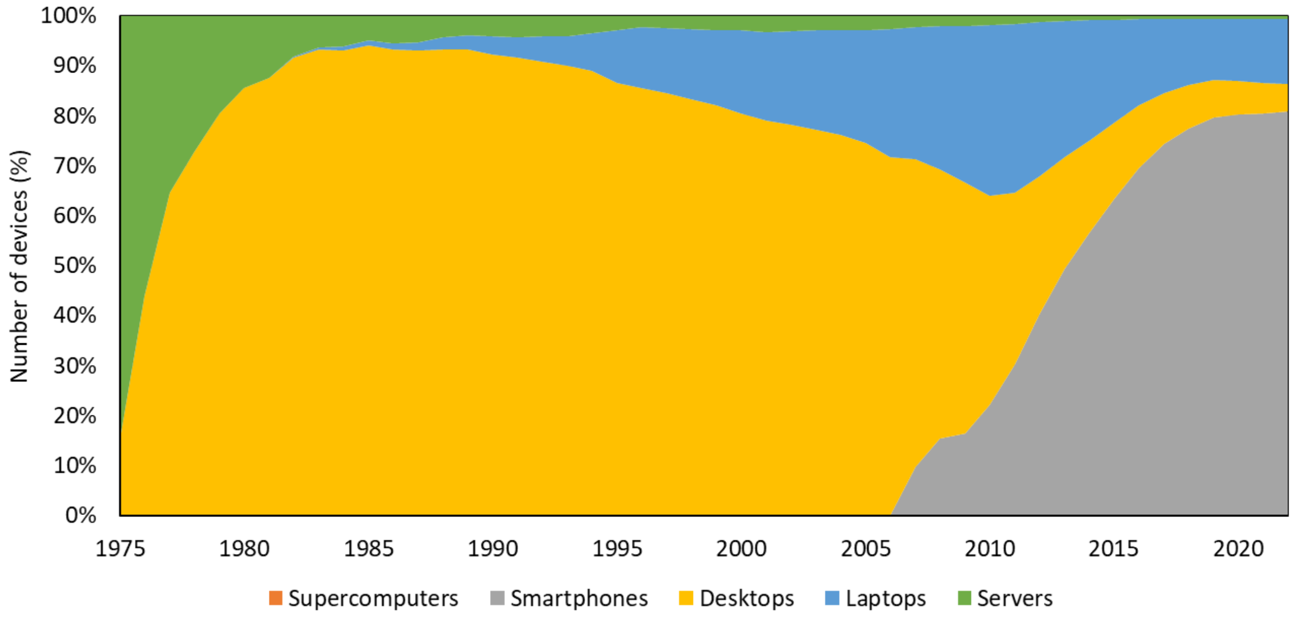

Figure S9 – Changes in the number of devices by type throughout the period studied.

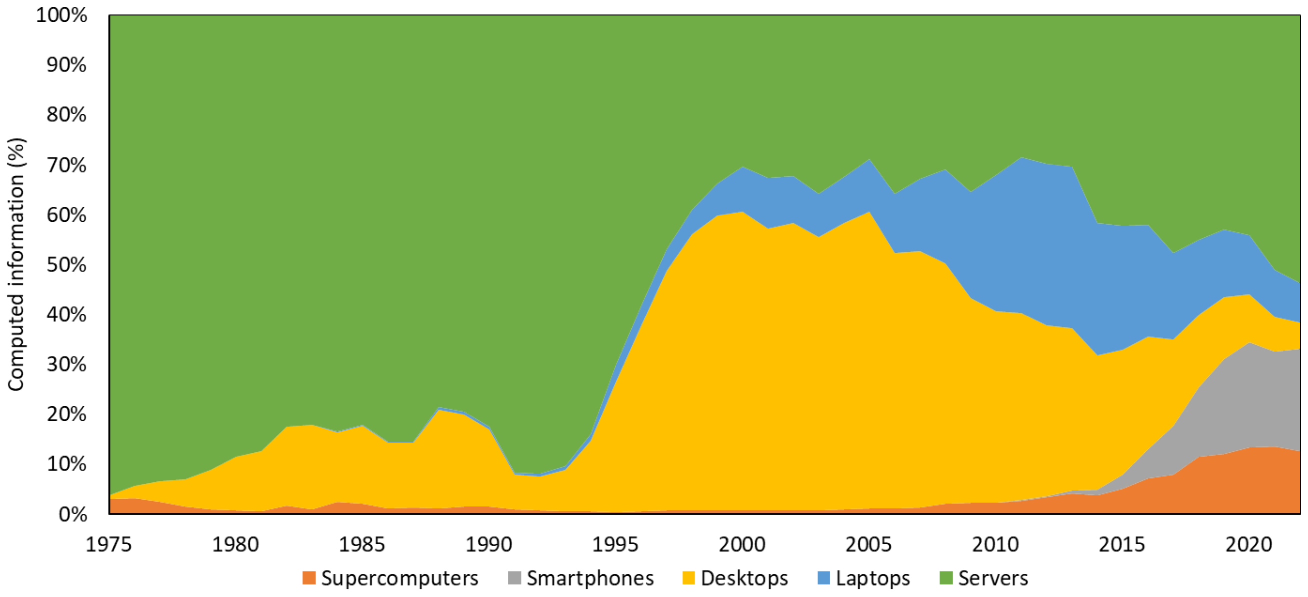

Figure S10 - Changes in the computed information share by type of device throughout the period studied.

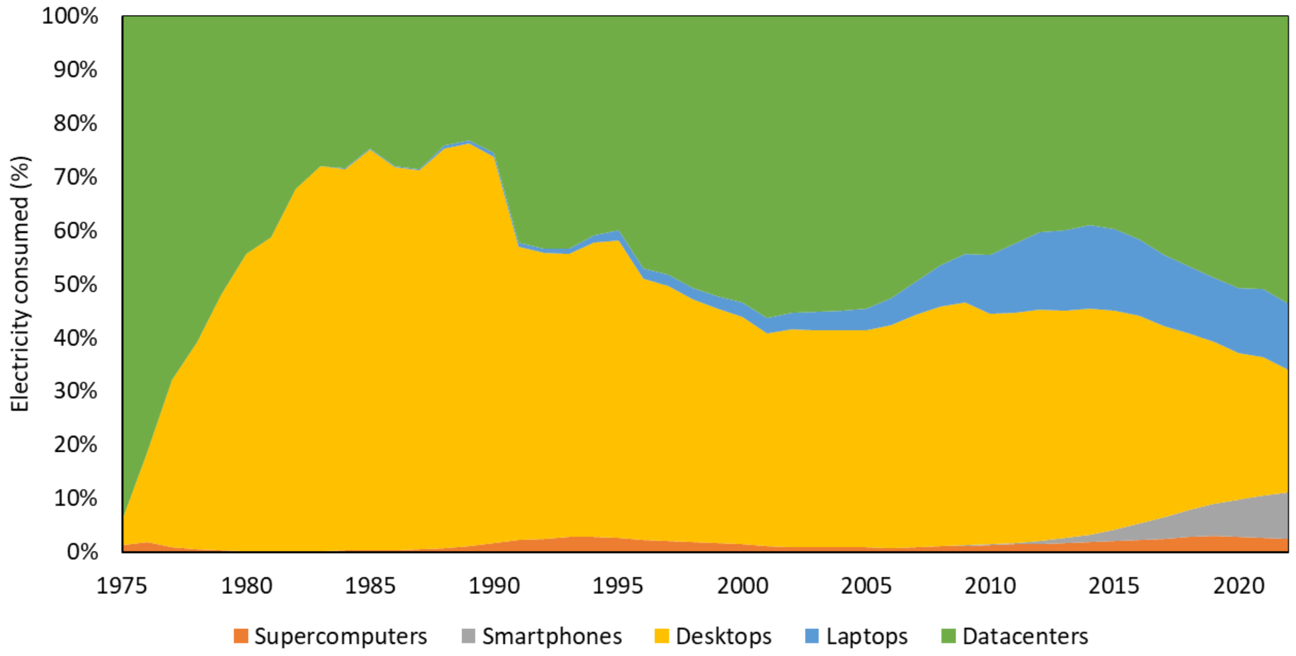

Figure S11 - Changes in the electricity consumption share by type of device throughout the period studied.

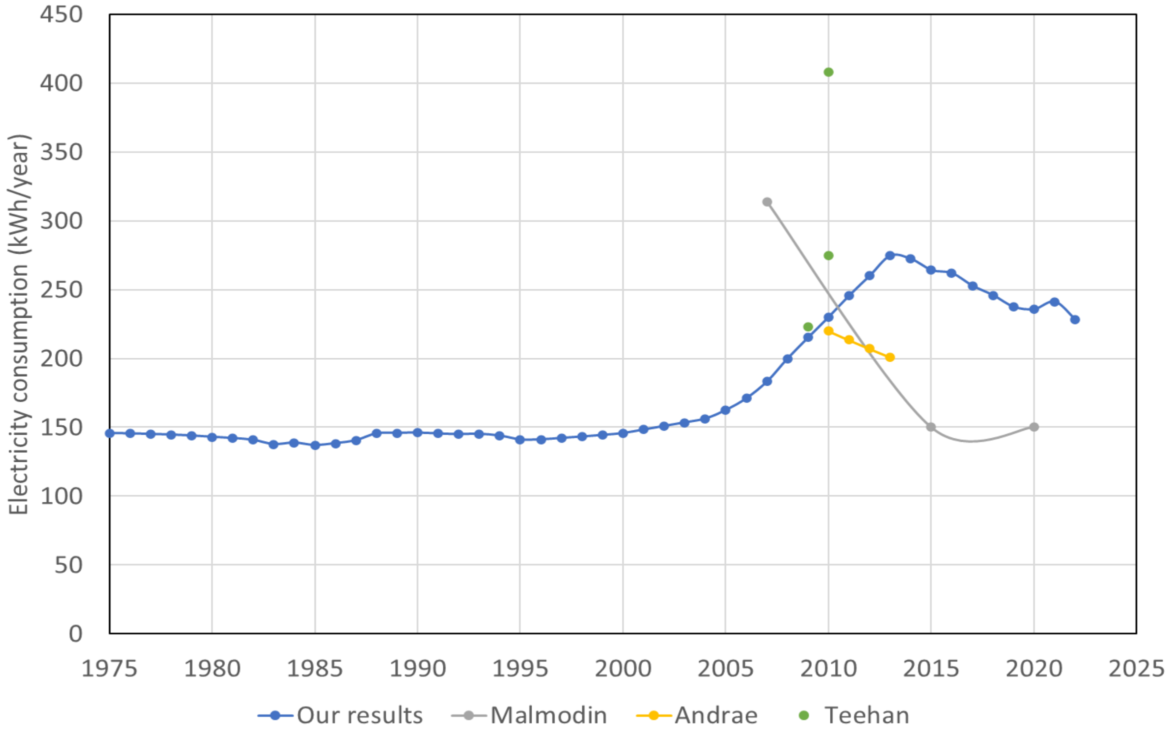

Figure S12 – Comparison of the annual consumption per desktop with the literature. Malmodin et al.[40–42], Andrae[43] and Teehan and Kandlikar[44]

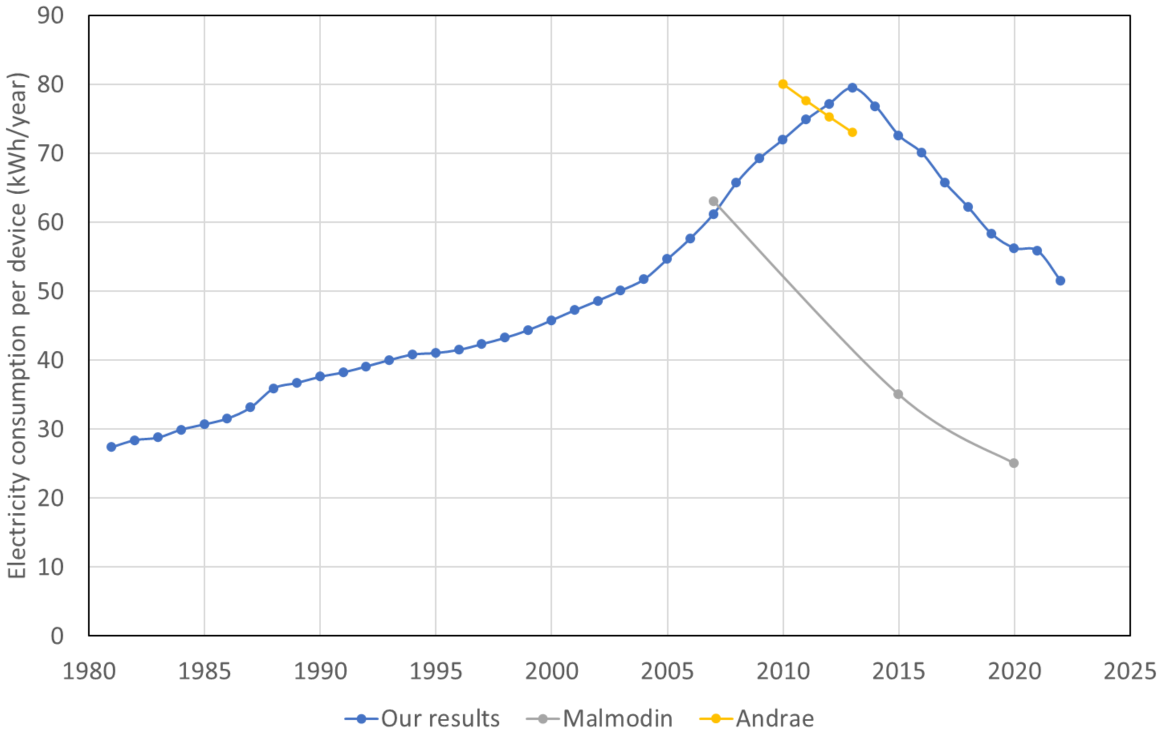

Figure S13 - Comparison of the annual consumption per laptop with the literature Malmodin et al.[40–42] and Andrae[43]

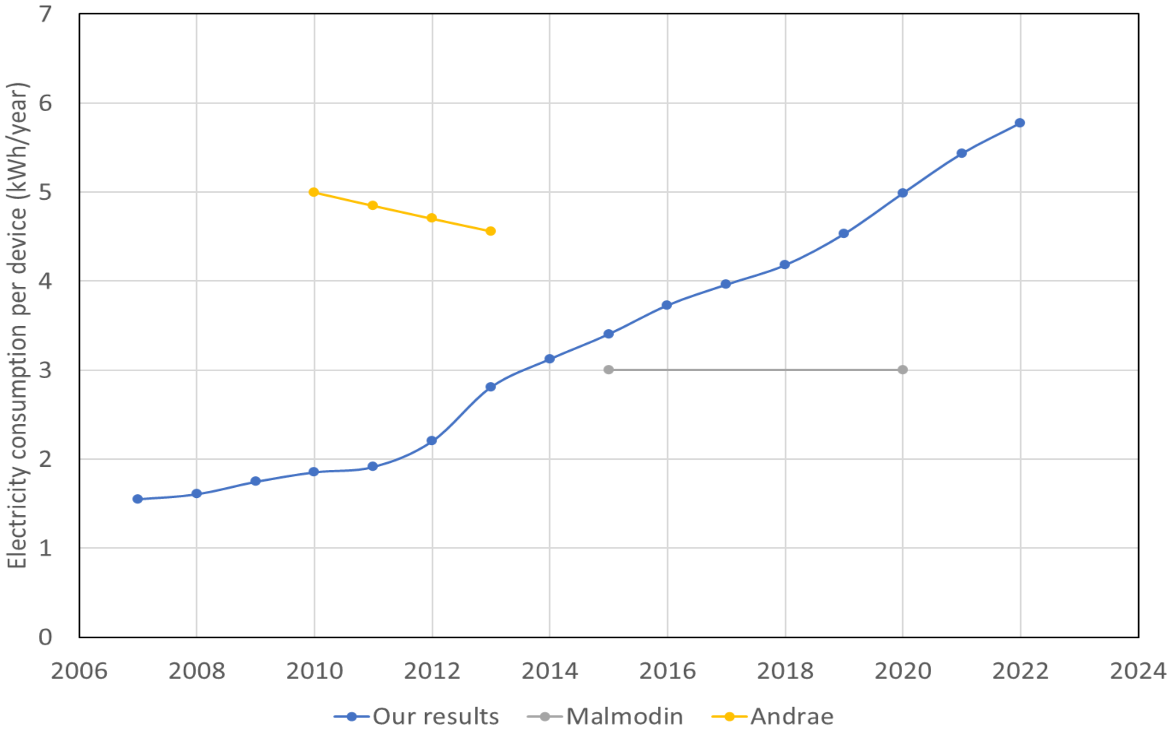

Figure S14 - Comparison of the annual consumption per smartphone with the literature Malmodin et al.[40,42] and Andrae[43]

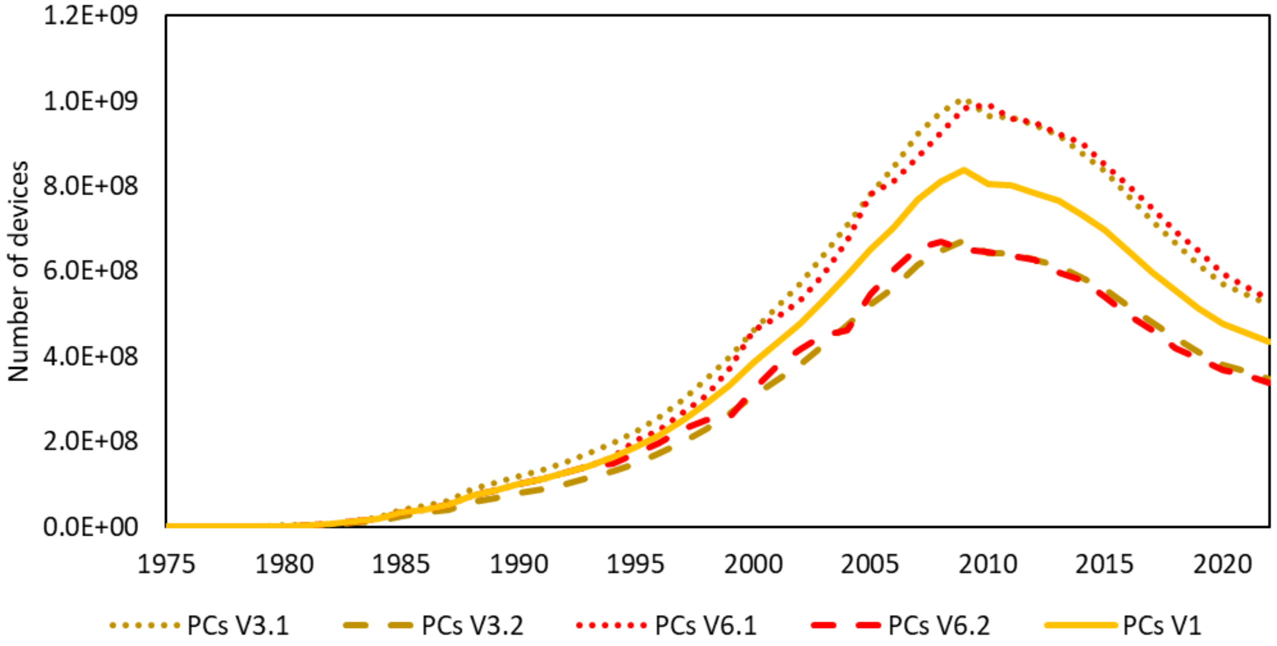

Figure S15 - Number of desktops in use throughout the study period. Different sensitivity analysis versions, compared to the main paper results shown by the line PCs V1.

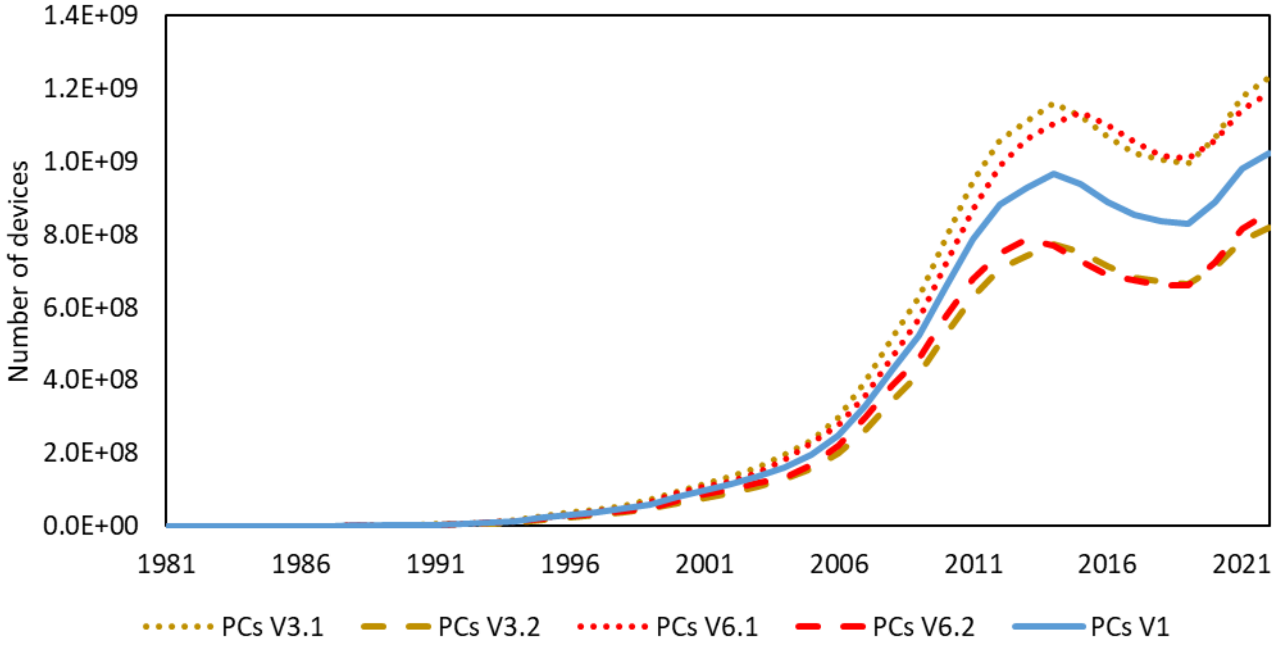

Figure S16 - Number of laptops in use throughout the study period. Different sensitivity analysis versions, compared to the main paper results shown by the line PCs V1.

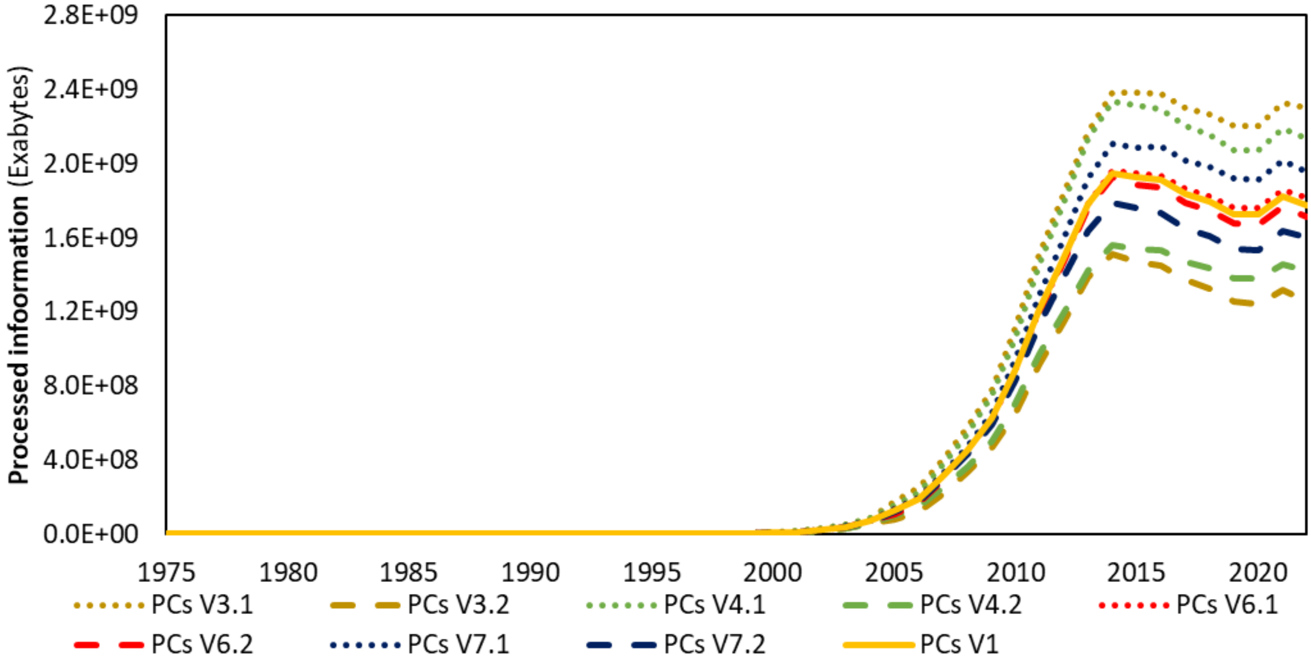

Figure S17 – Processed information by desktops throughout the study period. Different sensitivity analysis versions, compared to the main paper results shown by the line PCs V1.

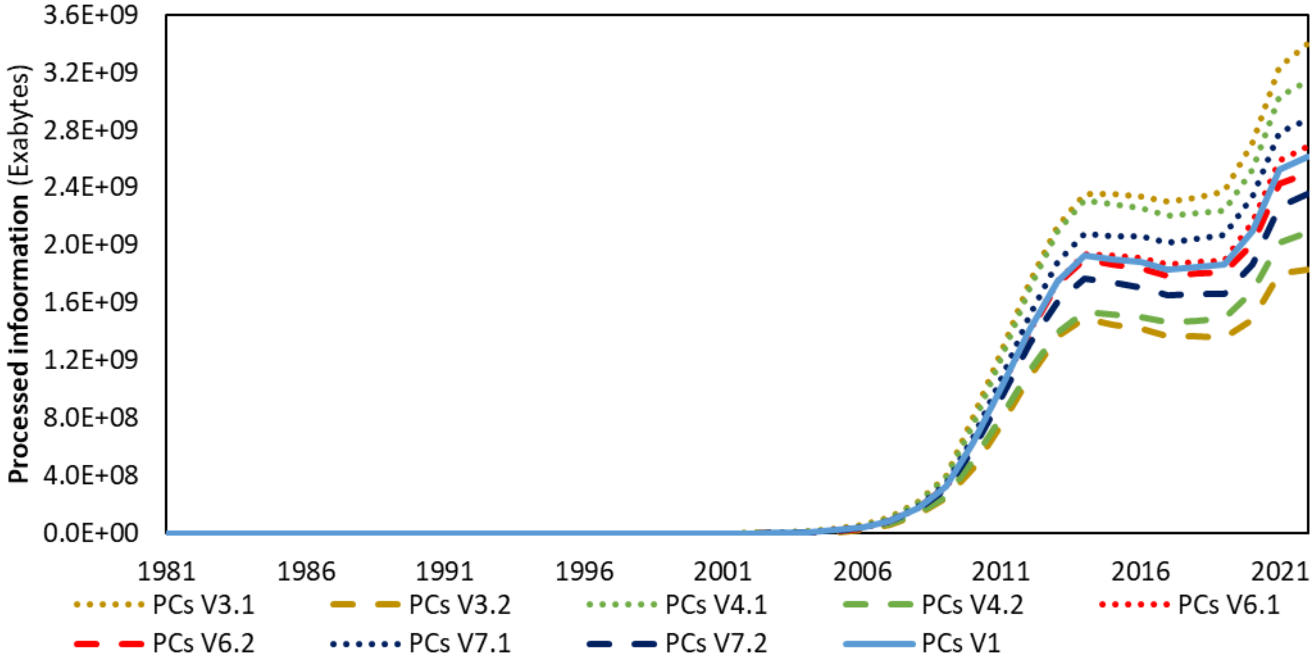

Figure S18 - Processed information by laptops throughout the study period. Different sensitivity analysis versions, compared to the main paper results shown by the line PCs V1.

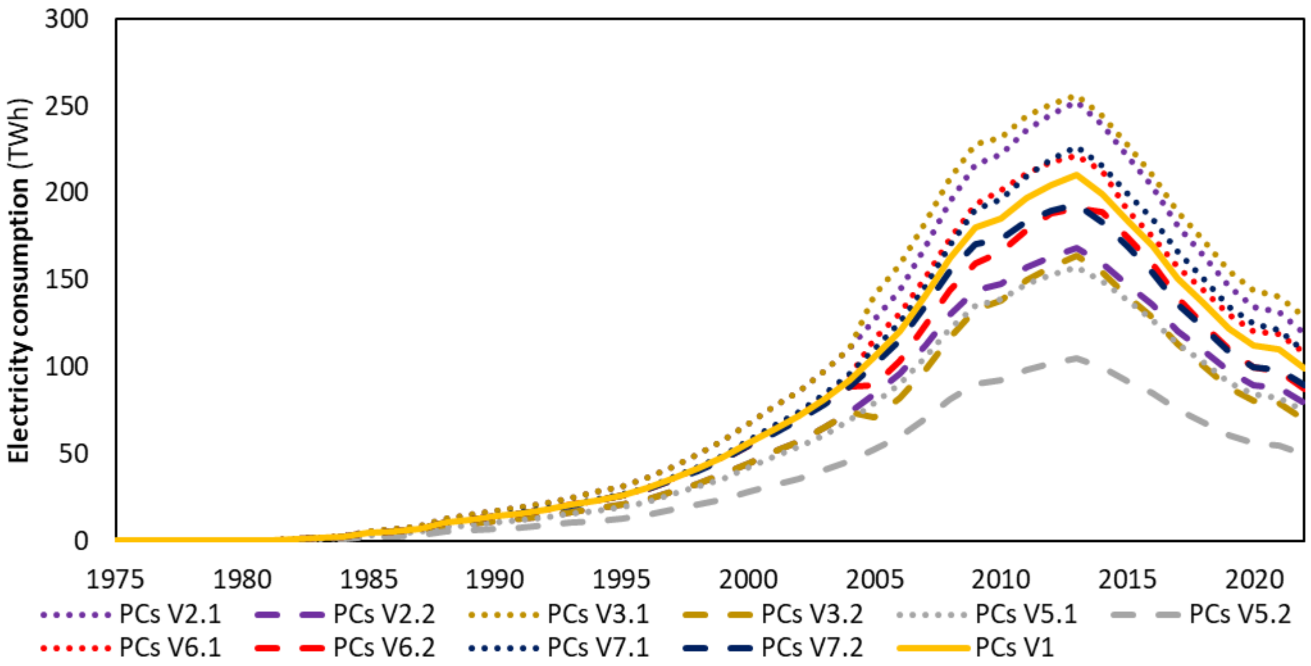

Figure S19 – Electricity consumed by desktops throughout the study period. Different sensitivity analysis versions, compared to the main paper results shown by the line PCs V1.

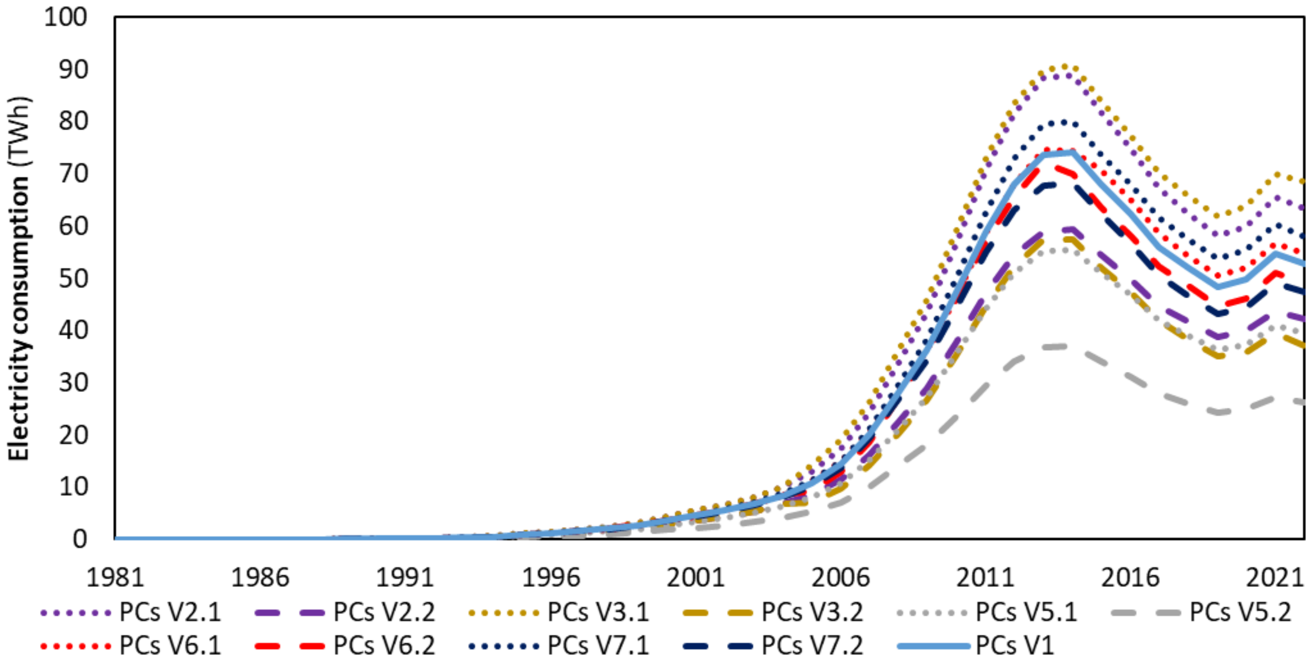

Figure S20 - Electricity consumed by laptops throughout the study period. Different sensitivity analysis versions, compared to the main paper results shown by the line PCs V1.

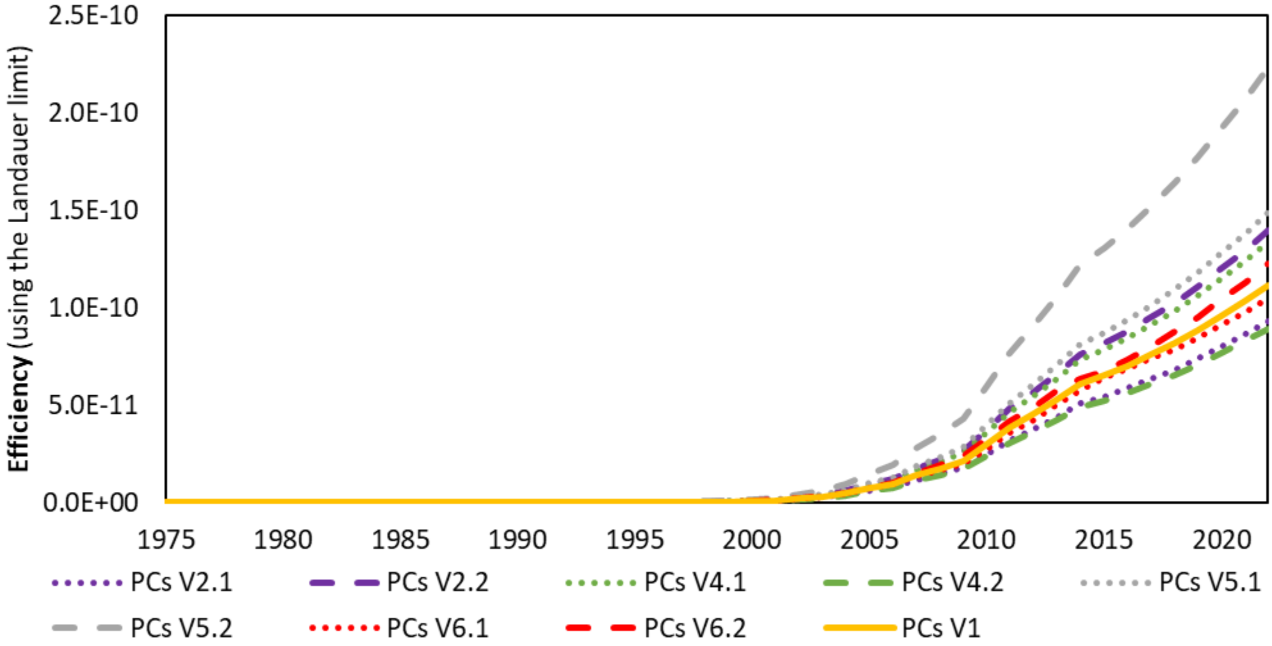

Figure S21 – Energy efficiency of desktops throughout the study period. Different sensitivity analysis versions, compared to the main paper results shown by the line PCs V1.

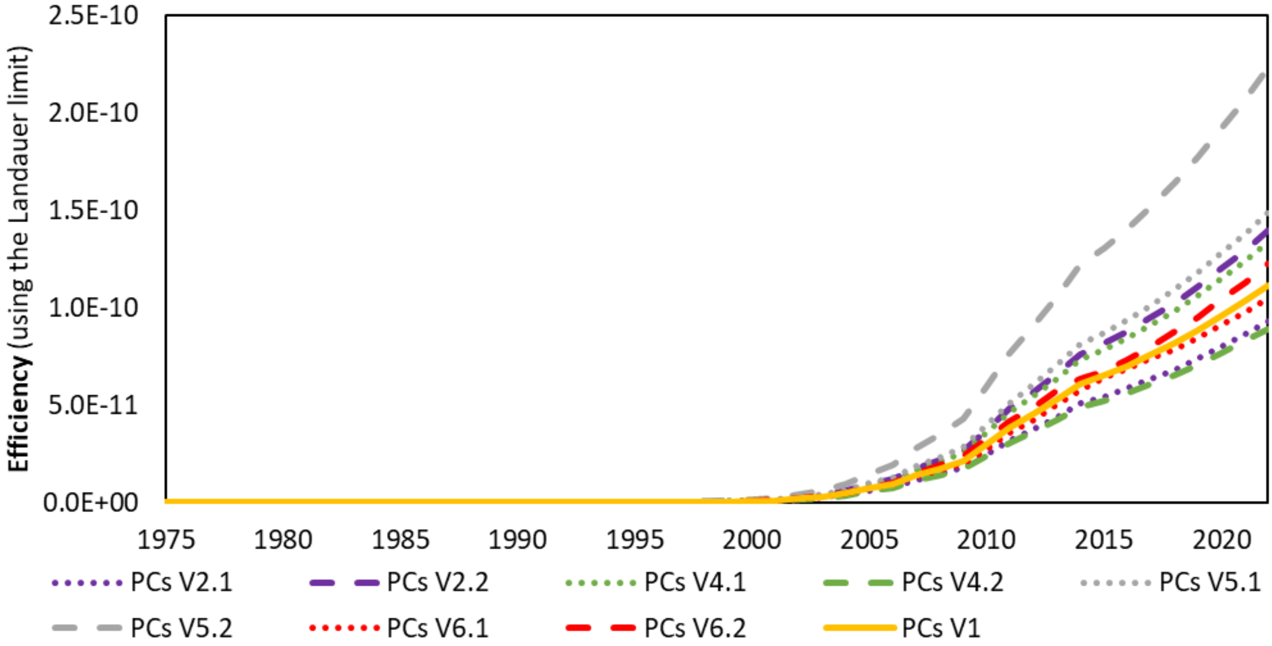

Figure S22 - Energy efficiency of laptops throughout the study period. Different sensitivity analysis versions, compared to the main paper results shown by the line PCs V1.

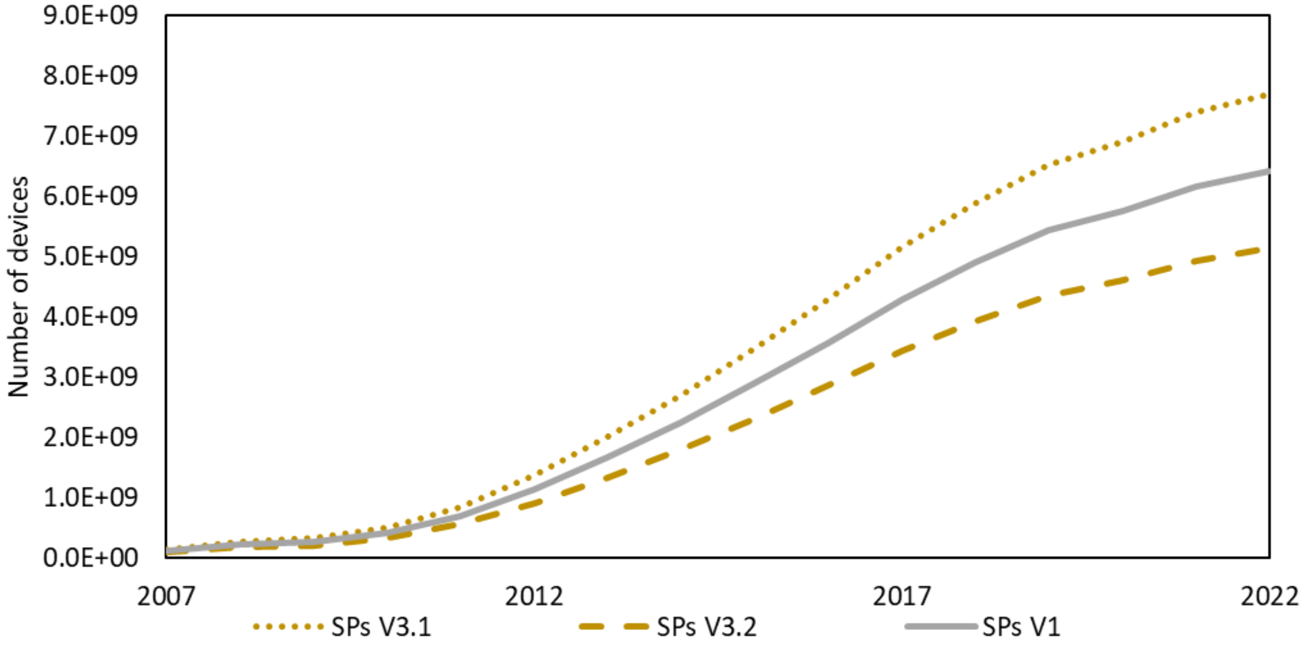

Figure S23 - Number of smartphones in use throughout the study period. Different sensitivity analysis versions, compared to the main paper results shown by the line SPs V1.

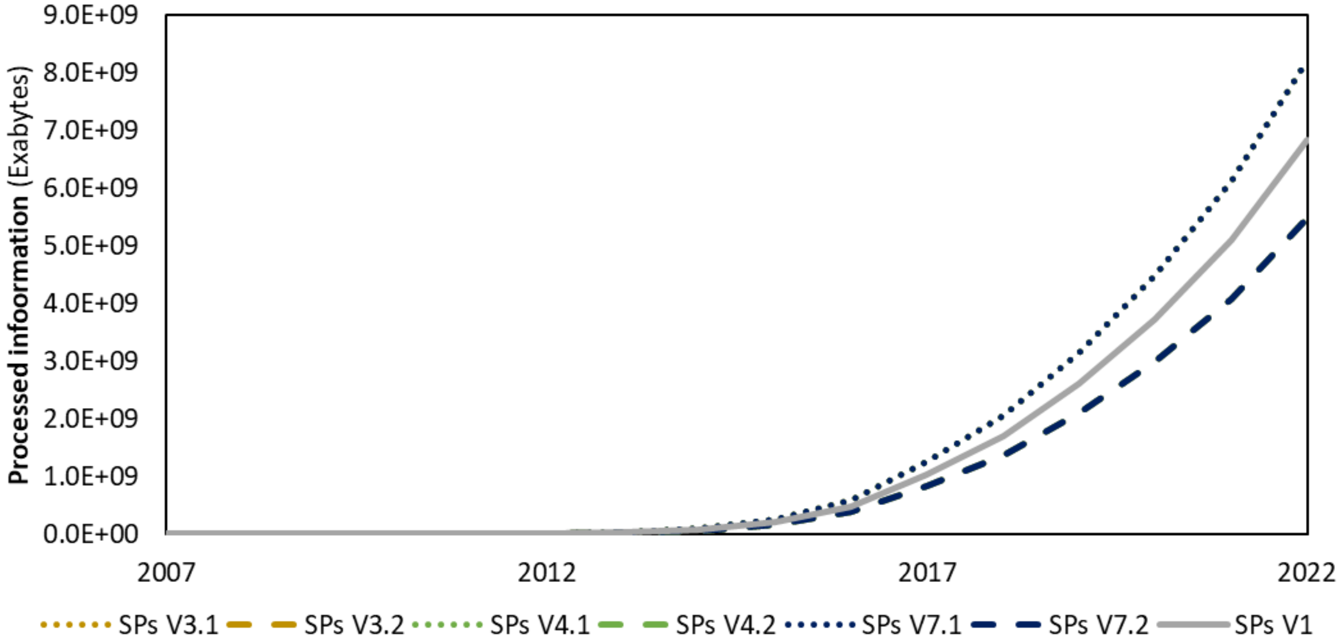

Figure S24 - Processed information by smartphones throughout the study period. Different sensitivity analysis versions, compared to the main paper results shown by the line SPs V1. Versions 3.1, 4.1 and 7.1 have the same results so do versions 3.2, 4.2 and 7.2 so they are on top of each other and are not visible in the figure.

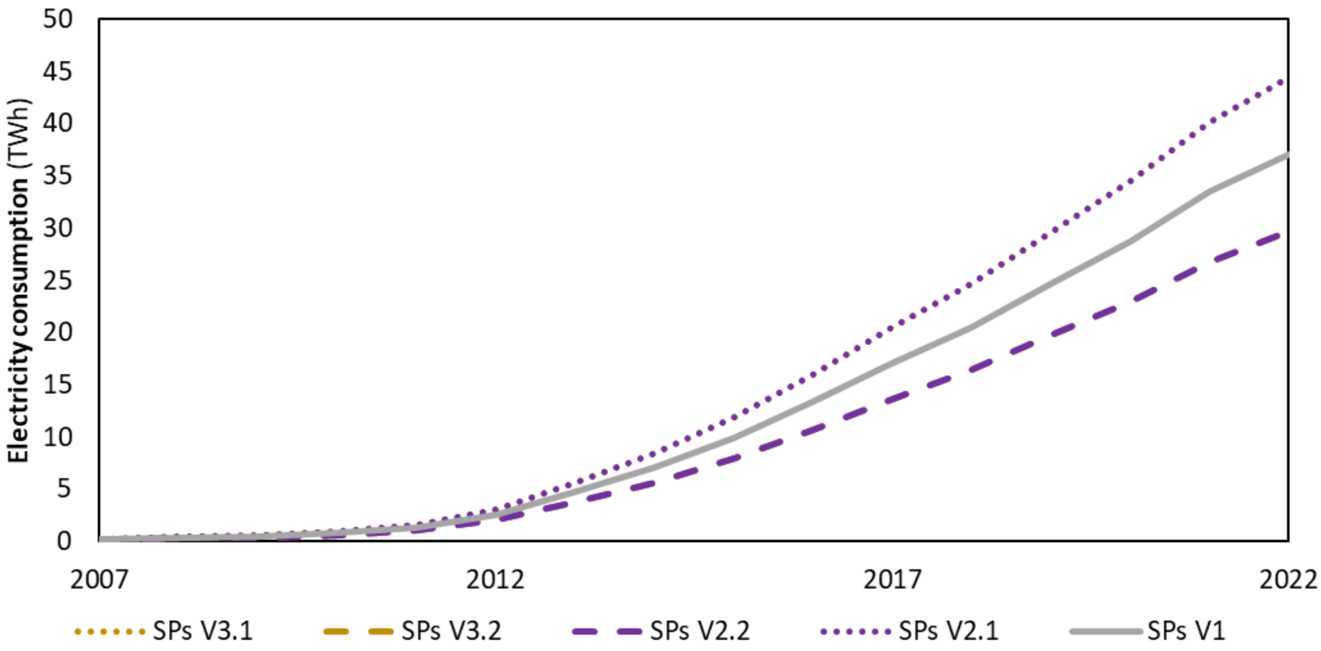

Figure S25 - Electricity consumed by smartphones throughout the study period. Different sensitivity analysis versions, compared to the main paper results shown by the line SPs V1. Versions 2.1 and 3.1 have the same results so do versions 2.2 and 3.2 so they are on top of each other and are not visible in the figure.

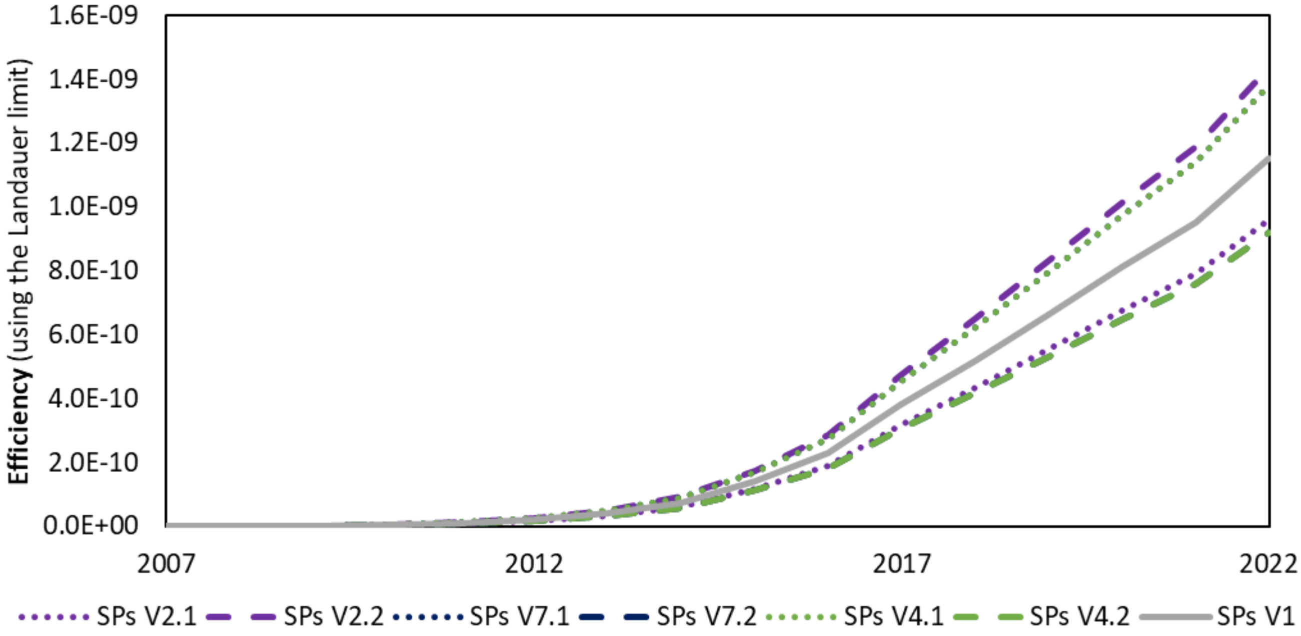

Figure S26 - Energy efficiency of smartphones throughout the study period. Different sensitivity analysis versions, compared to the main paper results shown by the line SPs V1. Versions 4.1 and 7.1 have the same results so do versions 4.2 and 7.2 so they are on top of each other and are not visible in the figure.

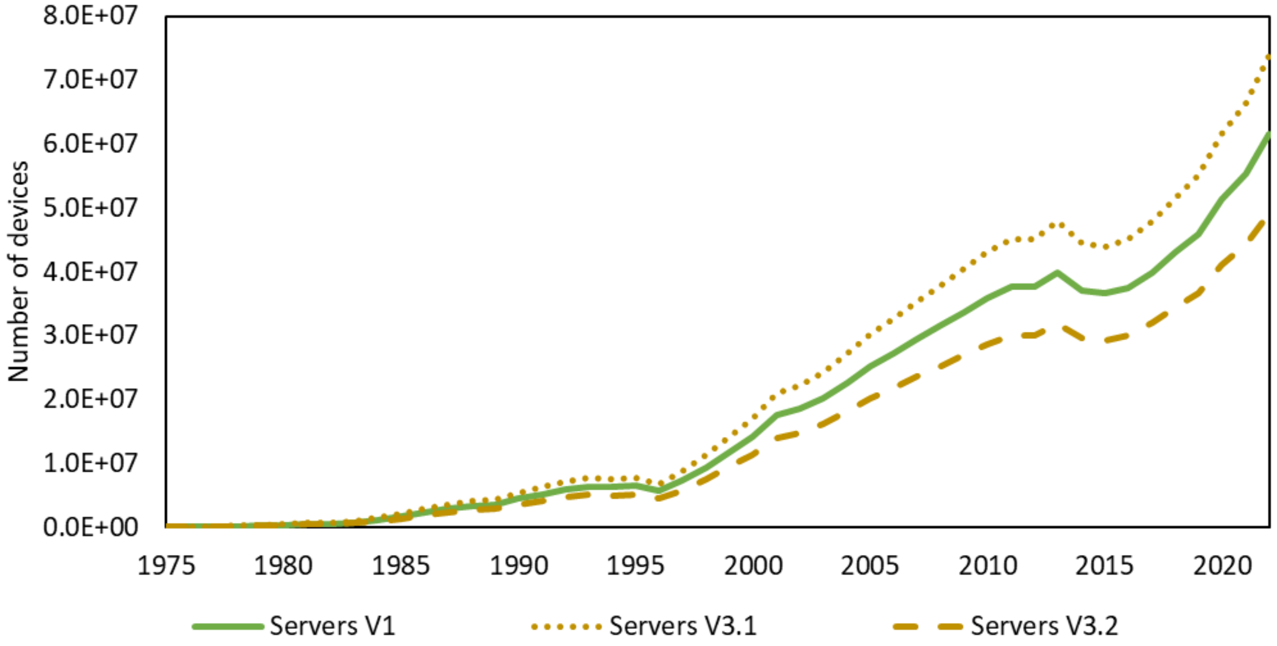

Figure S27 - Number of servers in use throughout the study period. Different sensitivity analysis versions, compared to the main paper results shown by the line Servers V1.

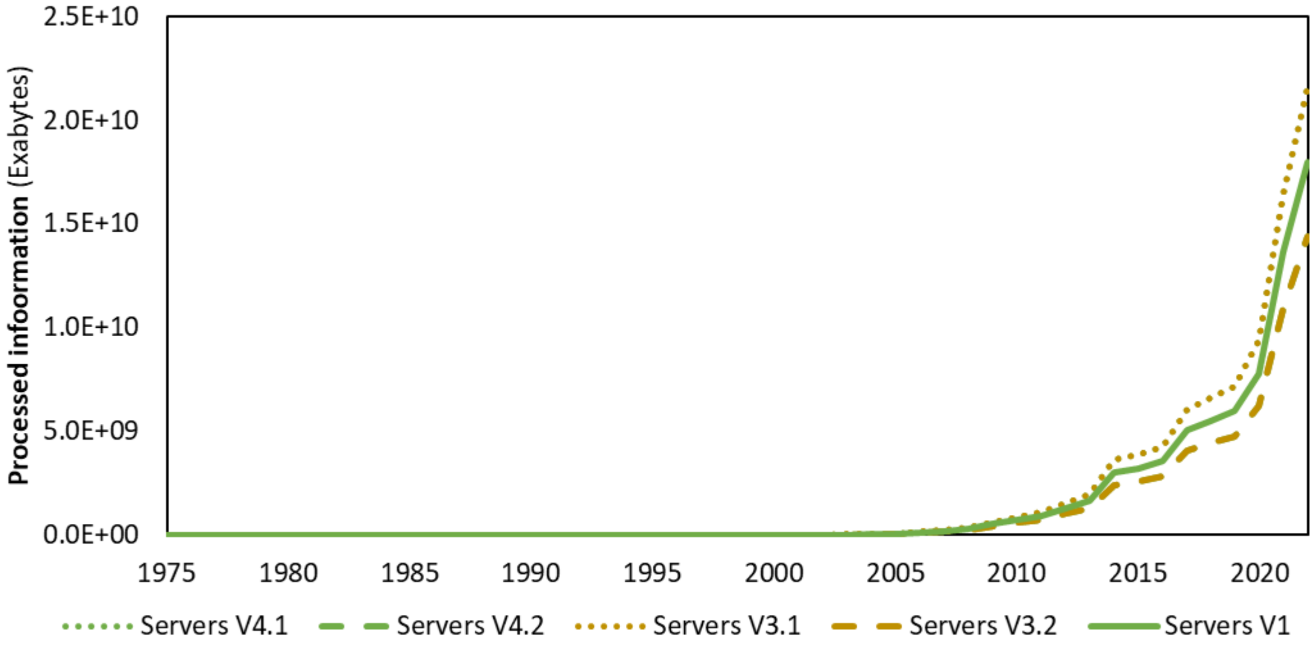

Figure S28 - Processed information by servers throughout the study period. Different sensitivity analysis versions, compared to the main paper results shown by the line SPs V1. Versions 3.1 and 4.1 have the same results so do versions 3.2 and 4.2 so they are on top of each other and are not visible in the figure.

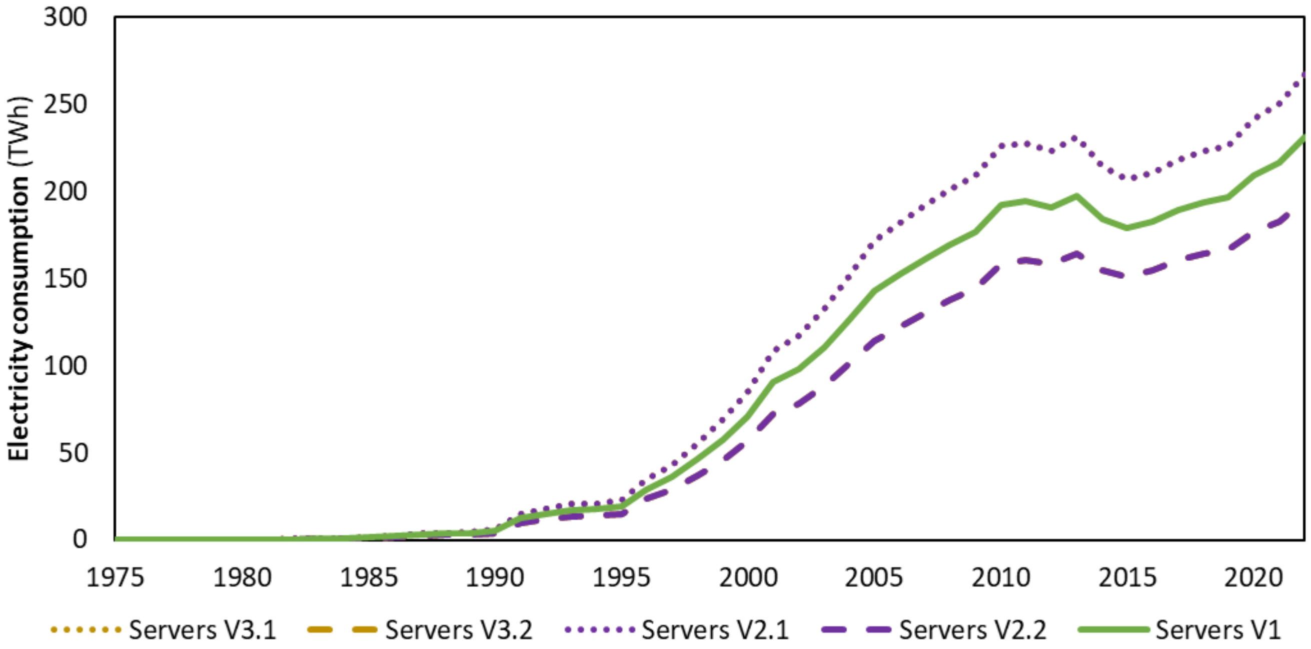

Figure S29 - Electricity consumed by datacentres throughout the study period. Different sensitivity analysis versions, compared to the main paper results shown by the line Servers V1. Versions 2.1 and 3.1 have the same results so do versions 2.2 and 3.2 so they are on top of each other and are not visible in the figure.

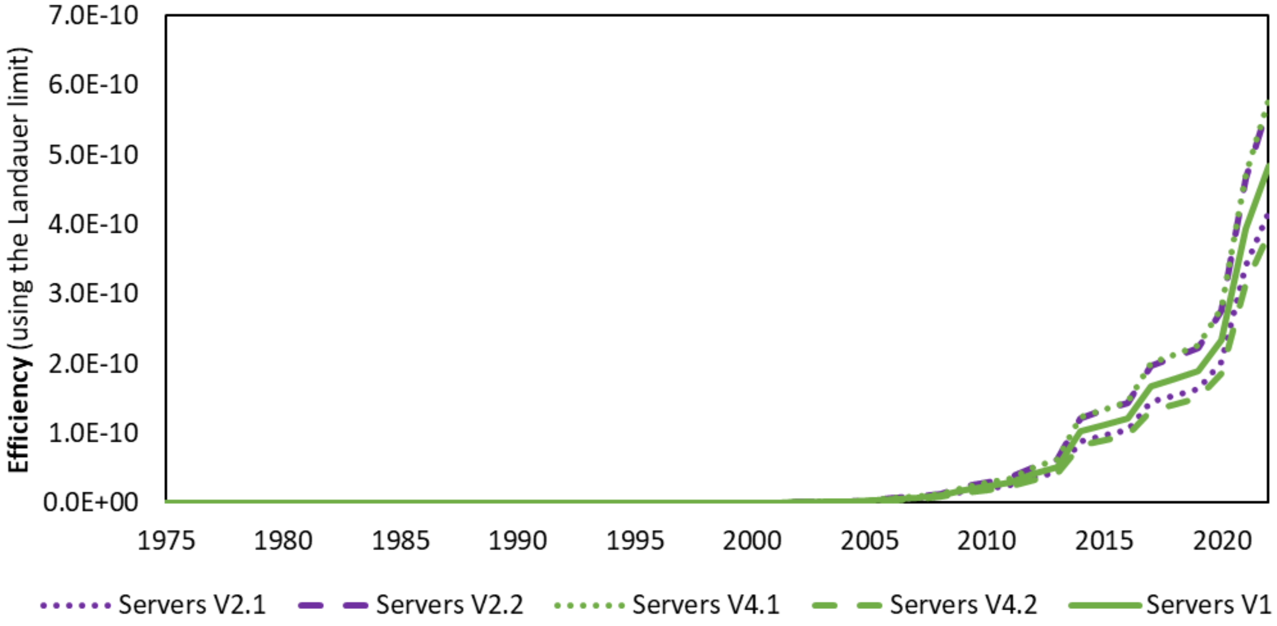

Figure S30 - Energy efficiency of datacentres throughout the study period. Different sensitivity analysis versions, compared to the main paper results shown by the line Servers V1.

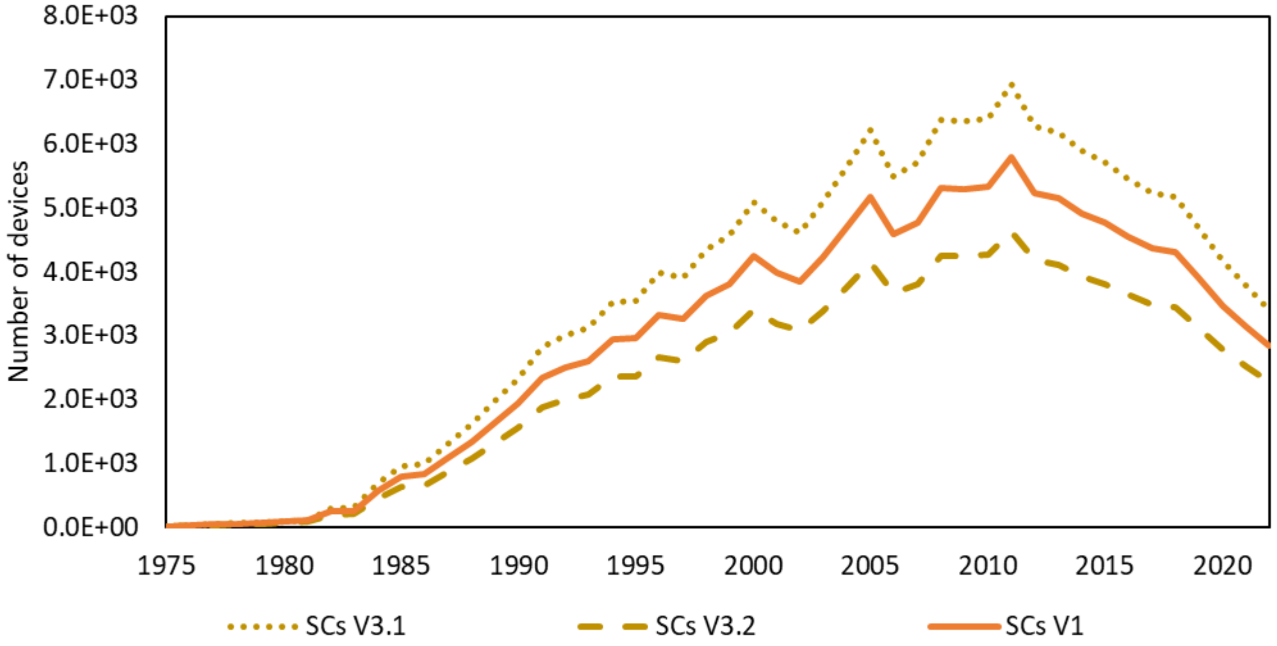

Figure S31 - Number of servers in use throughout the study period. Different sensitivity analysis versions, compared to the main paper results shown by the line SCs V1.

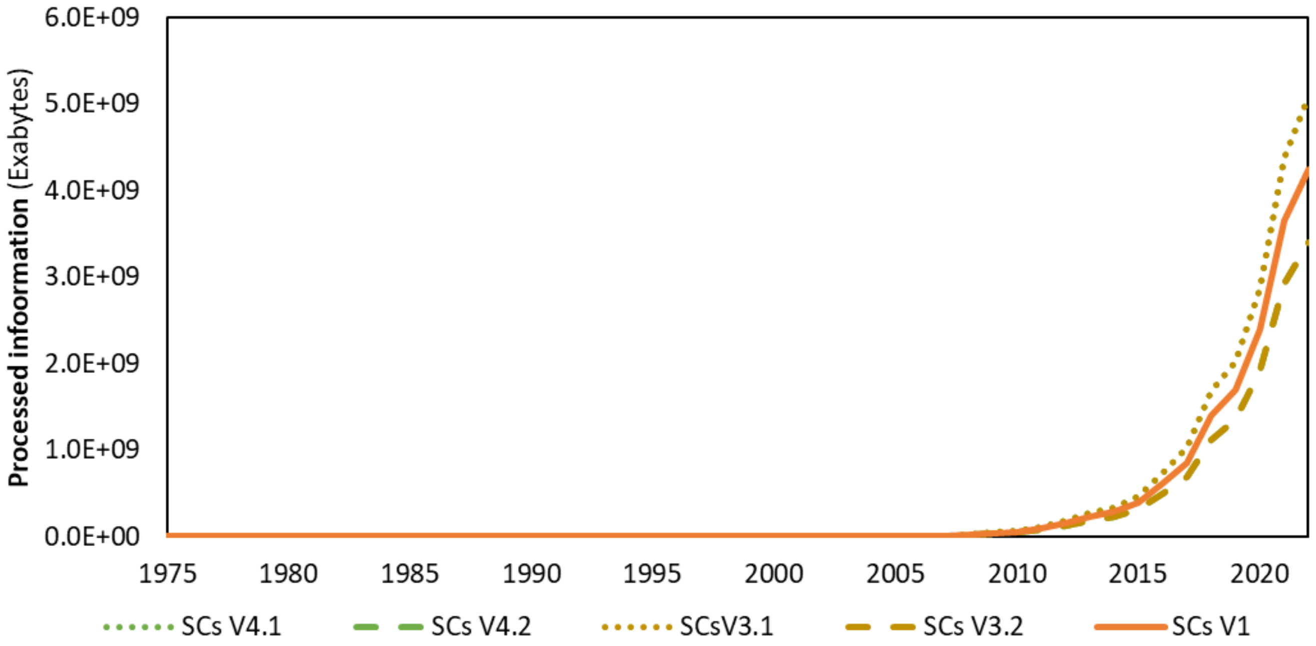

Figure S32 - Processed information by supercomputers throughout the study period. Different sensitivity analysis versions, compared to the main paper results shown by the line SCs V1. Versions 3.1 and 4.1 have the same results so do versions 3.2 and 4.2 so they are on top of each other and are not visible in the figure.

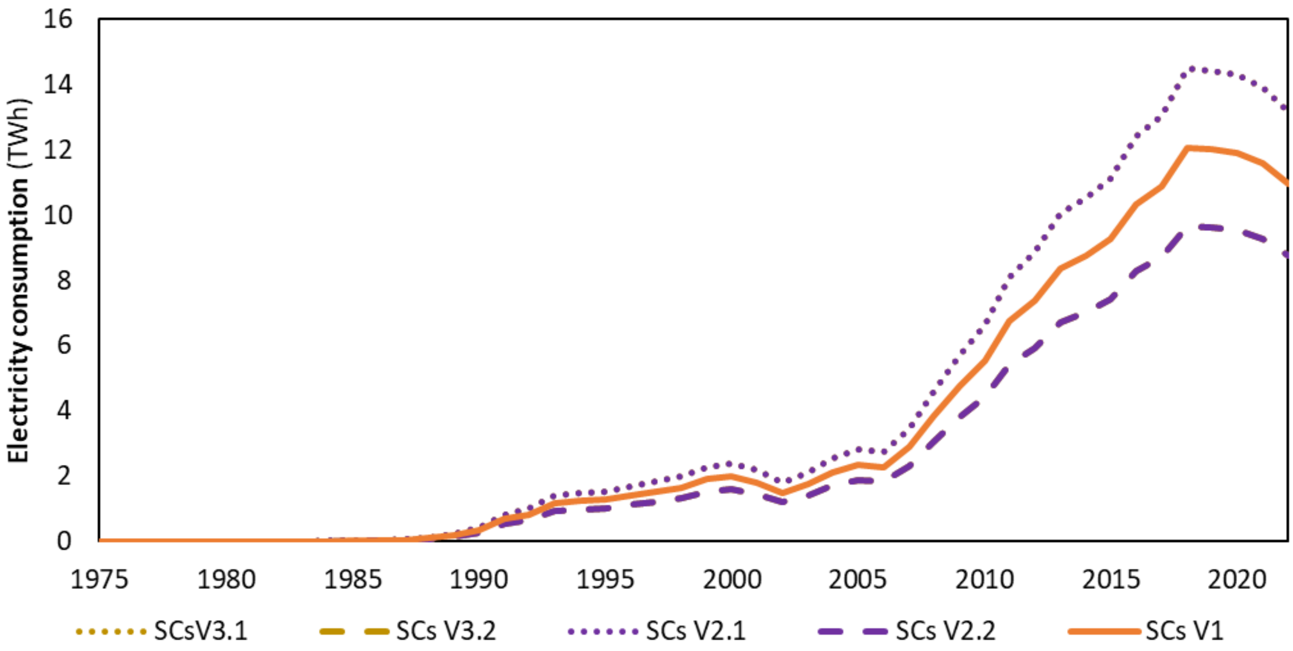

Figure S33 - Electricity consumed by supercomputers throughout the study period. Different sensitivity analysis versions, compared to the main paper results shown by the line SCs V1. Versions 2.1 and 3.1 have the same results so do versions 2.2 and 3.2 so they are on top of each other and are not visible in the figure.

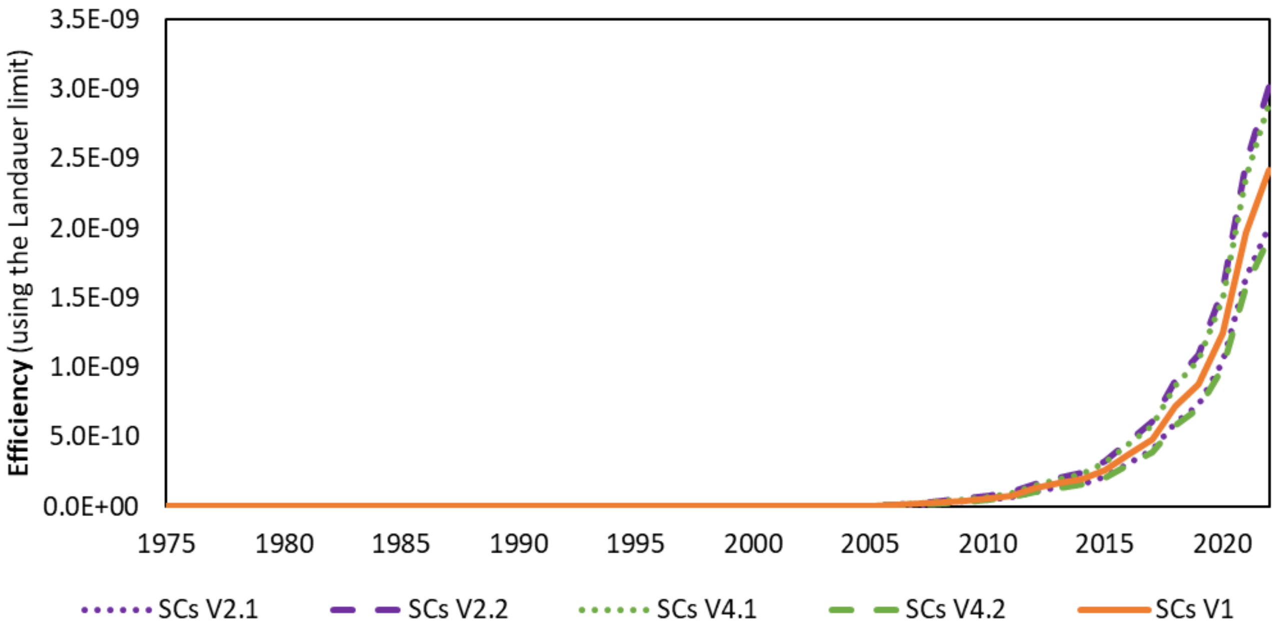

Figure S34 - Energy efficiency of datacentres throughout the study period. Different sensitivity analysis versions, compared to the main paper results shown by the line SCs V1.

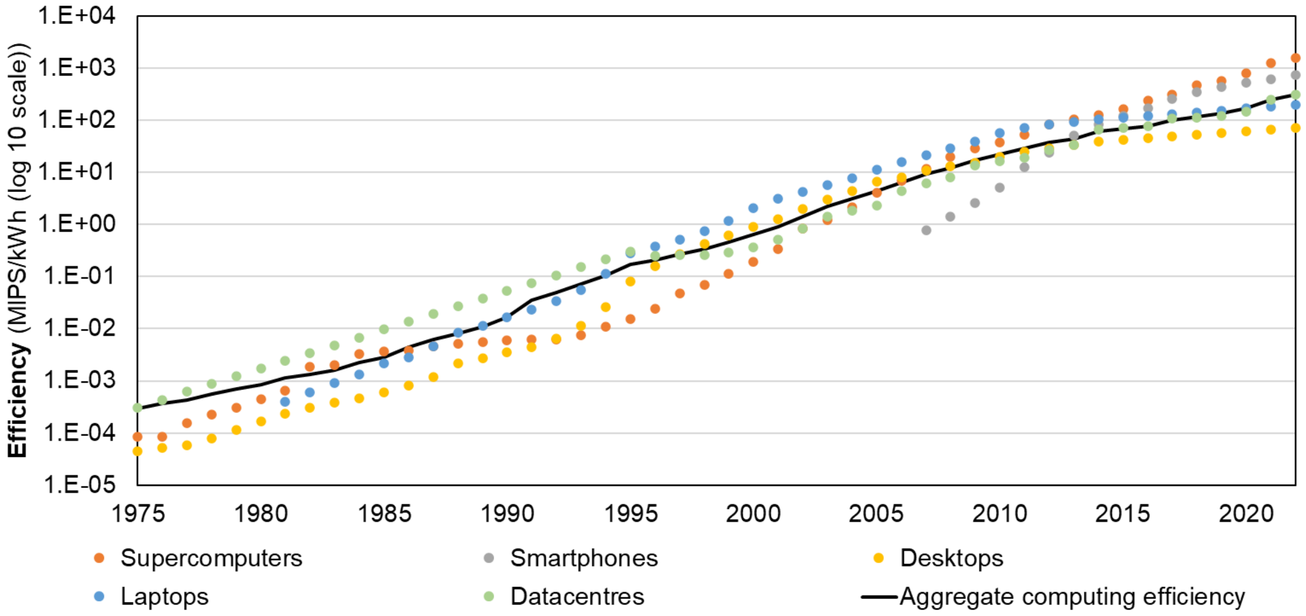

Figure S35 - Energy efficiency of computer estimated using the MIPS/kWh efficiency metric

Supplementary tables

Table S1 – Stocks comparison between our estimates and Hilbert and López[13] estimates, for servers and personal computers.

	Stocks			
	Servers		Personal Computers	
	Hilbert and López	Our estimate	Hilbert and López	Our estimate
1986	2 715 000	2 382 000	41 191 836	41 191 836
1993	11 222 000	6 419 000	151 930 586	151 930 586
2000	14 680 000	14 113 600	465 460 671	465 460 671
2007	33 977 000	29 416 002	1 034 024 833	1 098 987 694

Table S2 - Yearly hours of use comparison between our estimates and Hilbert and López[13] estimates, for servers and personal computers.

	Yearly hours of use			
	Servers		PCs	
	Hilbert and López	Our estimate	Hilbert and López	Our estimate
1986	2920	4380	52	1542
1993	2920	8760	263	1450
2000	2920	8760	343	1221
2007	2920	8760	583	1374

Table S3 - Average performance comparison between our estimates and Hilbert and López[13] estimates, for servers and personal computers.

	Average performance			
	Servers		PCs	
	Hilbert and López	Our estimate	Hilbert and López	Our estimate
1986	14.8	25.0	5.28	0.653
1993	170	399	40.2	11.0
2000	1130	1 840	749	917
2007	6532	33 581	5017	11171

Table S4 – Computed information comparison between our estimates and Hilbert and López[13] estimates, for servers and personal computers.

	Computed information					
	Total		Servers		PCs	
	Hilbert and López	Our estimate	Hilbert and López	Our estimate	Hilbert and López	Our estimate
1986	1.61E+07	3.45E+07	1.54E+07	2.98E+07	5.86E+05	4.67E+06
1993	3.95E+08	2.82E+09	3.02E+08	2.56E+09	8.56E+07	2.54E+08
2000	1.51E+10	8.53E+10	5.18E+09	2.60E+10	9.72E+09	5.90E+10
2007	4.27E+11	3.00E+12	5.77E+10	9.88E+11	2.79E+11	1.98E+12

Table S5 – Comparison of the annual consumption per desktop with Malmodin et al.[40–42] and Andrae[43]. Annual consumption is calculated by multiplying hours of use by power consumed.

	Desktops				
	Hours of use	Power consumed (W)	Annual unit electricity consumption (kWh/year)		
	Our results			Malmodin	Andrae
2007	1374	133	183	314	-
2008	1481	135	200	-	-
2009	1570	137	215	-	-
2010	1653	139	230	-	220
2011	1740	141	246	-	213
2012	1816	143	260	-	207
2013	1891	145	275	-	201
2014	1848	147	272	-	-
2015	1769	149	264	150	-
2016	1731	151	262	-	-
2017	1648	153	253	-	-
2018	1582	155	246	-	-
2019	1506	158	237	-	-
2020	1477	160	236	150	-

Table S6 - Comparison of the annual consumption per laptop with Malmodin et al.[40–42] and Andrae[43]. Annual consumption is calculated by multiplying hours of use by power consumed.

	Laptops				
	Hours of use	Power consumed (W)	Annual unit electricity consumption (kWh/year)		
	Our results			Malmodin	Andrae
2007	1374	44	61	22	-
2008	1481	44	66	-	-
2009	1570	44	69	-	-
2010	1653	44	72	-	80
2011	1740	43	75	-	79
2012	1816	42	77	-	80
2013	1891	42	79	-	84
2014	1848	42	77	-	-
2015	1769	41	73	34	-
2016	1731	40	70	-	-
2017	1648	40	66	-	-
2018	1582	39	62	-	-
2019	1506	39	58	-	-
2020	1477	38	56	29	-

Table S7 – Summary of the sensitivity analysis made for personal computer (desktops and laptops). Each column shows a parameter that was tested, and the rows show the name of the version.

	**Personal computer (PCs)**					
			Usage time (hours)			
Version	Useful life (years)	Number of devices	for home pcs	for office pcs	Power data	Performance
Version 1	See section 1.4					
Version 2.1	Equal to version 1				20% more than version 1	Equal to version 1
Version 2.2	Equal to version 1				20% less than version 1	Equal to version 1
Version 3.1	Equal to version 1	20% more than version 1	Equal to version 1		Equal to version 1	
Version 3.2	Equal to version 1	20% less than version 1	Equal to version 1		Equal to version 1	
Version 4.1	Equal to version 1					20% more than version 1
Version 4.2	Equal to version 1					20% less than version 1
Version 5.1	Equal to version 1				0.375 of rated power	Equal to version 1
Version 5.2	Equal to version 1				0.25 of rated power	Equal to version 1
Version 6.1	6 after 1990	Equal to version 1				
Version 6.2	4 after 1990	Equal to version 1				
Version 7.1	Equal to version 1		20% more than version 1	Equal to version 1	Equal to version 1	
Version 7.2	Equal to version 1		20% less than version 1	Equal to version 1	Equal to version 1	

Table S8 - **Summary of the sensitivity analysis made for smartphones.** Each column shows a parameter that was tested, and the rows show the name of the version.

**Smartphones**					
Version	Useful life (years)	Number of devices	Usage time (hours)	Annual unit energy	Performance
Version 1	See section 1.5				
Version 2.1	Equal to version 1			20% more than version 1	Equal to version 1
Version 2.2	Equal to version 1			20% less than version 1	Equal to version 1
Version 3.1	Equal to version 1	20% more than version 1	Equal to version 1	Equal to version 1	
Version 3.2	Equal to version 1	20% less than version 1	Equal to version 1	Equal to version 1	
Version 4.1	Equal to version 1				20% more than version 1
Version 4.2	Equal to version 1				20% less than version 1
Version 7.1	Equal to version 1		20% more than version 1	Equal to version 1	
Version 7.2	Equal to version 1		20% less than version 1	Equal to version 2	

Table S9 - Summary of the sensitivity analysis made for servers. Each column shows a parameter that was tested, and the rows show the name of the version.

<table>
<tr><th></th><th colspan="5">Servers</th></tr>
<tr><th>Version</th><th>Useful life (years)</th><th>Number of devices</th><th>Usage time (hours)</th><th>Power data</th><th>Performance</th></tr>
<tr><td>Version 1</td><td colspan="5">See section 1.6</td></tr>
<tr><td>Version 2.1</td><td colspan="3">Equal to version 1</td><td>20% more than version 1</td><td>Equal to version 1</td></tr>
<tr><td>Version 2.2</td><td colspan="3">Equal to version 1</td><td>20% less than version 1</td><td>Equal to version 1</td></tr>
<tr><td>Version 3.1</td><td>Equal to version 1</td><td>20% more than version 1</td><td>Equal to version 1</td><td colspan="2">Equal to version 1</td></tr>
<tr><td>Version 3.2</td><td>Equal to version 1</td><td>20% less than version 1</td><td>Equal to version 1</td><td colspan="2">Equal to version 1</td></tr>
<tr><td>Version 4.1</td><td colspan="4">Equal to version 1</td><td>20% more than version 1</td></tr>
<tr><td>Version 4.2</td><td colspan="4">Equal to version 1</td><td>20% less than version 1</td></tr>
</table>

Table S10 - Summary of the sensitivity analysis made for supercomputers. Each column shows a parameter that was tested, and the rows show the name of the version.

	**Supercomputers**			
	Useful life (years)	Number of devices	Energy consumption	Performance
Version 1	See section 1.3			
Version 2.1	Equal to version 1		Estimated using an increase of 20% in power intensity	Equal to version 1
Version 2.2	Equal to version 1		Estimated using an decrease of 20% in power intensity	Equal to version 1
Version 3.1	Equal to version 1	20% more than version 1	Equal to version 1	Equal to version 1
Version 3.2	Equal to version 1	20% less than version 1	Equal to version 1	Equal to version 1
Version 4.1	Equal to version 1			20% more than version 1
Version 4.2	Equal to version 1			20% less than version 1

Data/Methods S1 – Detailed methodology

This methodological description will start with a general outline of how we estimated information and electricity use. Afterwards, we detail the specific methods, including specific schematic representations, and references for each category of devices (supercomputers, personal computers, smartphones and servers). This first part is divided into two sections: information methods and energy methods. If you already have read the methods section of the paper, you can skip this section.

Note that descriptive statistics (e.g., mean, variance) are provided for rated power consumption values (see the supplementary data file). In contrast, stock and annual usage time are historical estimates obtained directly from literature rather than sampled variables, and therefore do not possess an underlying statistical distribution from which such statistics could be meaningfully derived. All raw values, assumptions, and sources are made fully available to ensure transparency and reproducibility.

Information Methods

Information processed by computers was estimated using the method summarized in Figure S1. We can divide this method into five steps.

Step 1 is the estimation of the stock of computers, also called installed base, for each year, using the computer lifetime (i.e. the average number of years a computer is in use), and the number of computers installed each year. The computer lifetime was based on literature and varied across different categories of computers. The number of computers installed per year was obtained from the literature and from specialized computer publications. The computer stock in a specific year was estimated by summing the new computers installed in that year to the stock of the previous year while subtracting the obsolete computers, computers that have exceeded their expected lifespan (i.e., the number of computers installed in year i-n, where n is the expected lifespan). Equation 1 summarizes this calculation.

$$Computer\ stock_i = Computer\ stock_{i-1} + new\ computers_i - obsolete\ computers_i \quad (1)$$

Step 2 is the calculation of the average performance of new computers (the number of instructions computers process per second) introduced in year i. We collected performance data for various computers, within each category. Some of these data points were taken from historical studies[1,2]. We complemented these data with publicly available performance data from different Standard Performance Evaluation Corporation (SPEC) benchmarks, as well as performance data from the Geekbench 6 and LINPACK benchmarks. The values obtained from these benchmarks were then used to estimate performance values in million instructions per second (MIPS). We then calculated the average performance of new computers for each year and used interpolation for the years where data was unavailable.

Step 3 is the calculation of the weighted average performance (WAP) of the stock of computers in year i. This step is necessary because the weighted average computer performance for a given year reflects the average performance of the current installed base, including all computers in use during that year. We calculated the Weighted Average Performance (WAP) for year i by multiplying the number of computers in stock from each installation year, by the average

performance of new computers for that year and repeating this process for all computers in stock. Finally, we divided this value by the total computer stock. Equation 2 summarizes this calculation where n is the number of installation years.

$$Weighted\ average\ performance\ (WAP)_i = \frac{\sum_{j-n+1}^{j} average\ performance_j \times\ No.of\ new\ computers_j}{Computer\ stock_i} \quad (2)$$

Step 4 is the calculation of the number of computations for each year. This was done by multiplying the WAP, the computer stock and the average time of computer use in each year, as shown in equation 3. The average time of computer use varied depending on the type of computer and changed annually for some categories.

$$No.of\ computations_i = WAP_i\ \times Computer\ Stock_i\ \times average\ time\ of\ computer\ use_i \quad (3)$$

Finally, step 5 is the conversion from computations to bits. This conversion was made using a conversion factor that varied over the years and depended on the word length of computers. Essentially, we multiplied the result of equation 3 by this conversion factor.

Energy Methods

The electricity used by computers was estimated using the method summarised in Figure S2, which is divided into 4 steps.

Step 1, which estimates the stock of computers for each year is the same as step 1 in the information methodology.

Step 2 is the calculation of the average power of new computers in year *i*. This step is analogous to step 2 from the information method , with the difference being the use of power data instead of performance data.

Step 3 is the calculation of the weighted average power of the stock, which is like step 3 of the information methodology, with the difference being the use of power data instead of performance data, as shown in equation 4.

$$Weighted\ average\ power_i = \frac{\sum_{i-n+1}^{i} average\ power\ _j \times\ No.of\ installed\ computers_j}{Computer\ stock_i} \quad (4)$$

Finally, step 4 is the estimation of electricity consumption. We estimated the electricity used by computers in each year of our study by multiplying the weighted average power of the stock of computers, the average hours of use, and the number of computers in use.

The calculation of electricity consumption was based on various assumptions regarding the share or type of power that should be used (rated power, peak power, active power). These assumptions depended on the availability of power data and type of computer. For example, for PCs, we assumed 50 % of rated power based on previous studies[3,4].

Supercomputers

Supercomputers are computer systems that use more than one central processing unit (CPU) to process information; these systems are built to achieve high levels of performance enabling them to solve difficult computational problems. There are various architectures that can be used to create a supercomputer, currently the highest performance supercomputers belong to two different categories: clusters and massive parallel processing (MPP) systems. Clusters are defined as "a parallel computer system comprising an integrated collection of independent nodes, each of which is a system in its own right, capable of independent operation"[5]. In contrast, the term MPP originally referred to systems that operated in parallel while sharing components, such as memory [5]. However, most modern MPP systems are now described as "distributed-memory system composed of multiprocessor shared-memory nodes" [6].

In this section, we describe the methods and references used to calculate information processed and electricity consumption of supercomputers, **Figure S*3*** and **Figure S*4*** are the supercomputer specific versions of Figure S1 and Figure S2.

Data about supercomputers was mostly obtained from TOP500[7]. TOP 500 data, which includes performance information (measured in FLOPS), is published twice a year, in June and November, since 1993. Each list comprises the 500 most powerful, best performing in the LINPACK benchmark[8], non-distributed supercomputers. TOP 500 lists also have data about power consumption but only for a small number of supercomputers. Since the TOP 500 lists only had power data for a reduced number of supercomputers prior to 2005, we looked for additional information. We retrieved a table with more data for supercomputers power from the National Center for Atmospheric Research (NCAR)[9]. The supercomputers in that table enabled an expansion of the number of data points for the years between 1989 and 2004.

The total number of new supercomputers installed each year, step 1b of Figure S3, was obtained using TOP500 lists through the following method. For instance, for 2010, we counted in the November 2010 list the number of supercomputers that had 2010 as their installation year. We used this number as the first estimation of supercomputers for that year. Since the list changes during the year, with new supercomputers being added, and others dropping off, we made the following corrections to the initial value. First, we added the computers that appeared in the June 2010 list, that had an installation year of 2010 and a performance below the minimum performance of the last supercomputer considered in the November 2010 list (these computers were excluded from the November 2010 list but had 2010 as the date of installation). Then, we added the supercomputers with installation year of 2010 that appear in the June 2011 list but do not appear in the November 2010 list. We applied this method to estimate the number of supercomputers installed each year between 1993 and 2022, also referred to as the number of new supercomputers per year. The performance values in these lists are in floating point operations per second (FLOPS). We convert them to instructions per second (IPS) by using the ratio 1 MFLOPS = 3 MIPS, based on the conversion factor in Giladi (1996)[10].

The next step, step 2a, involved calculating the total performance added each year, by summing the performance of all supercomputers.

To calculate the number of supercomputers in use, we first needed to estimate their useful life, step 1a of Figure S3. This estimation assumes that a supercomputer loses its usefulness when its performance matches that of the best personal computer (PC). In other words, the useful life of a supercomputer ends when a PC can process the same amount of information. Thus, the useful life of all supercomputers from year k is determined as the difference between i and k, where i represents the year when the performance of the best PC equalled that of the average

supercomputer in year k. This assumption that all supercomputers of year k reach the end of their useful life in year i, is a necessary simplification to avoid the need of an individual evaluation of each supercomputer. Table S16 shows the useful life of supercomputers by year of installation.

Step 1 involved calculating the number of supercomputers in use each year, also mentioned as supercomputer stock or installed base. This was done by summing the number of supercomputers still within their useful life (equation 1). For example, to calculate the number of supercomputers in use in 2013, we summed all the supercomputers installed between 2002 and 2013.

Afterwards, in step 2, the average performance of new supercomputers in a given year was calculated by dividing the total performance added that year (step 2a) by the number of supercomputers installed during the same period (step 1b). Average performance for each year is available in Table S17.

Average power was calculated in the same way, with the difference being that power data was only available for a reduced number of supercomputers (between 10% and 65% of all the new installed supercomputers, after 2005). As a result, the average power obtained was calculated based solely on the supercomputers for which power data was available. An additional problem with power data was that during the early years of the study period very few data points were available. To address this issue, we interpolated between the years with data. The average power value obtained was assumed as a proxy for the average power use of all supercomputers installed in a given year and was used to calculate their electricity consumption.

Step 3 involved estimating the weighted average performance (WAP) of all supercomputers in use in a specific year. This step followed the same method explained in step 3 of the general methods (equation 2).

In step 4, we estimated the total number of computations supercomputers can make each year (equation 5) assuming that supercomputers work 90% of the time in a year. This step followed the method explained in step 4 of the general method (equation 3).

$$No. of\ computations_i = WAP_i \times Stock_i \times 365 \times 24 \times 60 \times 60 \times 0.9 \quad (5)$$

Finally, in step 5, we converted computations into bits by using a factor of 64, meaning that each computation or instruction corresponds to 64 bits. This choice was based on the use of double precision in the LINPACK benchmark[8,11]. A schematic representation of the method used to estimate information computed per year can be seen in Figure S3.

In contrast to performance data, which was available for all supercomputers, power data on the TOP 500 lists was only available for a relatively small number of supercomputers. It was only after 2005 that power data became available for more than 10% of the new supercomputers. Therefore, as explained above, we supplemented the power consumption data with information retrieved from NCAR[9]. Due to the limited availability and high variability of power data (see Table S35), we used an indirect method to obtain a more reliable estimate of electricity use. This approach uses power data indirectly by dividing the performance of each supercomputer by its power consumption, a metric commonly referred to as energy efficiency in specialized

publications like TOP500. By inverting this measure, we derived power intensity (W/MIPS) and calculated the average power intensity of supercomputers installed in year i, as outlined in step 1 of Figure S4.

In Step 2, we calculated the total power (TP) of the supercomputer stock for each year (equation 6).

$$Total\ power\ (TP)_i = \\ = \sum_{i-n+1}^{i} average\ power\ intensity_j \times total\ performance_j \tag{6}$$

To estimate electricity use in year *i*, step 3, the total power (TP) of the supercomputer stock of year i by the maximum possible computations in that year (calculated before when we were estimating information computed) and by the seconds a supercomputer is used in a year (equation 7). A schematic representation of this indirect method used to estimate electricity consumed per year can be seen in Figure S4.

$$Energy\ consumed \\ = TP \times 365 \times 24 \times 60 \times 60 \times 0.9 \tag{7}$$

Personal computers

This paper includes laptops, desktops and workstations in the personal computers (PCs) category. Previously, similar types of computers were referred to as microcomputers and minicomputers, and they are also included in this category. When we reference desktops throughout the text below, we are considering desktops and workstations put together. A schematic representation of the method used to estimate information processed by personal computers can be seen in Figure S1. Figure S2 is a schematic representation of the method used to estimate electricity consumed by personal computers.

Data about PCs was collected from several references. To calculate the installed base, step 1 in Figure S1, we used annual shipments from 2006 to 2022, obtained from Statista[12], and the installed base of PCs between 1985 and 2005 from Hilbert and López (2011). Prior to 1985 we relied on data obtained from Jeremy Reimer[14]. After 2005, we assumed a useful life of 5 years to calculate the installed base[13].

For estimating average power and performance (discussed below), we assumed a useful life based on Hilbert and López (2011): 5 years from 1990 onward, 7 years for 1987–1989, and 10 years for computers produced before 1986.

For a more detailed set of results, we divided PCs according to the place where they were used, home or office, and further separated them between laptops and desktops. This resulted in 4 different subcategories within PCs, each exhibiting varying characteristics, including differences in performance and power between laptops and desktops, as well as differing assumptions regarding hours of use for home and office PCs (Tables S18, S19 and S20). We estimated the stock and the number of new computers for each of the 4 subcategories (Table S21).

We began by estimating the number of laptops and desktops. After 2005, we used data from Statista[15–17] to calculate the number of desktops and laptops. Prior to 2005, we had a data point indicating that laptops had a 2% market share in 1986[18]. Between 1986 and 2005, we

interpolated the share of laptops and assumed that the remaining PCs were desktops. We also interpolated data between 1981 and 1986, assuming that in 1981 only 0.1% of all PCs were laptops. Before 1981, we assumed that no laptops existed, as the first laptop data we have indicates that they entered the market in that year.

To separate between home and office PCs, we calculated the number of home PCs after 2013 assuming that it was equal to the number of households with computers[19]. Between 2005 and 2012, we used the global share of households with computers from Statista and ITU[20]. Between 1982 and 1991, we had data for the number of new PCs in the USA, separated by home and office PCs[21], which we used as a proxy for the world. We assumed that in 1975, 1% of all new computers were home PCs, with the remaining 99% classified as office PCs. For the remaining years, we interpolated the share of new PCs from years with available data. The share of home PCs changed annually but was consistent across laptops and desktops. All the shares for each subcategory are available in Table S22.

Average performance of PCs, step 2 in Figure S1, was calculated separately for laptops and desktops by calculating the average of PCs with data available within each category. We collected performance data for individual PCs from previous studies[1,2]. We also calculated performance values, in MIPS, using publicly available performance data derived from benchmarks developed by the Standard Performance Evaluation Corporation (SPEC). Years with values of average performance that were either too low or too high when compared with neighbouring years were considered outliers and eliminated. Given the exponential growth trend in average performance, we calculated the logarithm of all the years with data after eliminating the outliers. We then linearly interpolated between these logarithmic values and exponentiated them to create two series of average performance: one for laptops and one for desktops.

After 2010, we used a different method to estimate average performance due to scarcity of data points, with no data point available after 2016. This method employed the Geekbench 6 benchmark[22], which had data for various central processing units (CPUs), for both laptops and desktops. The first step was to convert from Geekbench 6 results to million instructions per second (MIPS) using CPUs that had both MIPS and Geekbench values. We established a trendline from these values to perform the conversion. The second step was to collect Geekbench 6 data for a series of CPUs that fitted the following criteria: they were launched between 2010 and 2022, were either mobile (used in laptops) or desktop CPUs, and were either the best or the worst in their category (mobile or desktop) across different product lines (intel core i3, i5, i7 and i9, Pentium and Celeron and AMD Ryzen 3,5,7 and 9). In some years, only one datapoint was available for a given category and product line. All the values of performance of these CPUs were converted to MIPS and the average per year was calculated. We then applied a logarithm to these values and plotted them to obtain trendlines for laptops and desktops. Finally, we calculated the average performance for each year between 2010 and 2022 using the established trendlines and exponentiated the results. The values of average performance per year are shown in Table S18.

The weighted average performance, step 3 in Figure S1, for laptops and desktops was calculated using equation 2, as described in the general method.

Step 4 involved calculating the total number of instructions that PCs perform each year, which was done using equation 3. We assumed office PCs were used 8 hours per working day (40 hours per week, assuming 52 weeks in a year), while home PCs were used a variable number of hours.

Between 1986 and 2007, the hours of home PC use were retrieved from Hilbert and López[13]. After 2013, we assumed that the amount of time home PCs were used was equal to the hours of internet use on computers with data retrieved from Digital data[23]. For the years between 2007 and 2013, we interpolated between the values in those two years, while for the period from 1975 to 1986, we assumed an average of 15 minutes of use per week in 1975 for interpolation. Hours of PC use are available in Table S20.

Finally, to convert computation into bits, we used the word length parameter of the computers for which we had performance data. We calculated both the average and the weighted average of word length for each year (Table S23). We then multiplied the weighted average of word length by the number of computations to calculate the number of bits of information processed each year.

In contrast to supercomputers, the electricity consumption of PCs was estimated using a direct method, see Figure S2. This method involved multiplying weighted average power consumption by the number of PCs in use and by the number of hours PCs were used. The number of PCs in use and hours of use were already discussed above. Regarding power consumption, we collected data for rated power consumption for almost all the PCs we had performance data for (see Table S34). We calculated the average for each year and eliminated the outliers. We then created a trendline and used it to calculate average rated power for each year for both laptops and desktops (Table S19). Regarding laptops this trendline was only used until 2005, year when battery capacity of laptops starts to stabilize[24], after that we assumed the average power was decreasing with the same trend as the power of laptop processors after 2010 (Table S33). This reduction in power of the average computer is in line with trends in the reduction in size, thickness and weight of laptops[24]. Afterwards, we calculated weighted average power using equation 4. However, rated power is an overestimation of the actual consumption of PCs[25]: the correct value is 25 to 50% of the rated power. Therefore, based on Harris et al.[25], we assumed that actual power consumption was 50 % of the rated power, choosing the upper limit to have the worst-case scenario in terms of electricity consumption. The calculation of electricity consumed is summarized by equation 8. We performed this calculation for each of the 4 subcategories of PCs: home desktops, office desktops, home laptops and office desktops. Results for each subcategory and total electricity consumed are available in Table S24.

$$\begin{gathered} Energy\ consumed = \\ = Weighted\ average\ power \times 0.5 \times Nº\ of\ PCs\ \times Hours\ of\ use \end{gathered} \tag{8}$$

Smartphones

Smartphones are the most recent type of computing device addressed in this study. They are also the most ubiquitous with more than 6 billion in use during the last year of our study, 2022. Since it is hard to define when the first smartphone was released, we collected data starting in 2007, the year when the first iPhone was released.

As with the other types of devices, we needed information about the stock and annual shipments. We retrieved data from Ericsson[26] that had the values for the stock of smartphones since 2011. To estimate the stock between 2007 and 2010, we collected data about annual sales of smartphones from 2007 onwards from Statista[27] and assumed a useful life of 1.75 years (since we calculated useful life of smartphones to be 1.79 in 2011) between 2007 and 2010. For 2007, we assumed that the stock was equal to the number of smartphones sold that year. Stock

estimation was the first step to obtain information processed by smartphones, Figure S5 is a schematic representation of the method used.

Step 1a involved calculating the useful life of smartphones from 2011 onward. Knowing the annual smartphone sales and the stock for each year, we calculated the number of years of sales needed to match the stock. The results for the useful life of smartphones are presented in Table S25.

Afterwards, we collected data about smartphone performance with two different methods. Prior to 2016, most smartphones used CPUs with performance values in MIPS that were based solely on the clock rate[28]. By utilizing the clock rate, CPU model and the number of CPUs used, we were able to create a performance trend. After 2016, we had to use the Geekbench 6 benchmark[22] which had data for performance for various smartphones. As with PCs, we needed to convert the performance values of the Geekbench 6 benchmark to MIPS. To do this, we used smartphones that had both MIPS and Geekbench values, from which we established a trendline for conversion. After converting all the Geekbench 6 benchmark values to MIPS, we calculated the average performance of new smartphones in each year, as shown in step 2 of Figure S5 (Table S26).

Step 3 in Figure S5 involved calculating the weighted average performance of the stock of smartphones for each year, which was done using equation 2.

Subsequently we calculated the number of instructions smartphones can process per year using equation 3. The number of hours a smartphone is used varies annually. For 2007, we retrieved data from Hilbert and López[13], after 2013 (inclusive), we assumed that the hours of use were equal to the hours of internet use on mobiles with data retrieved from Digital data[23] (Table S27). Between 2007 and 2013, we interpolated between the values in those two years. Smartphones are computing devices, but they also do many other things unrelated to computing, primarily related to communication. Therefore, in order to have an accurate estimate of instructions we tried to separate the different functions. Carroll and Heiser[29] tested a smartphone and presented results for electricity consumption of the main components. Using the data collected about hours of internet use and the use pattern from Carrol and Heiser previous study[30], we estimated that a little more that 10% of the electricity was used by the CPU. With that value in mind, and incorporating a safe margin of error, we assumed that smartphones only computed 20% of the hours of internet use in mobiles. It is important to note that we always assume that smartphones (and all other computing devices) are computing at peak performance meaning that our assumption is equivalent to having the smartphone computing at 20% of the peak capacity during the total hours estimated initially.

The final step, step 5, involved converting instruction to bits. We used the number of bits of the instruction set of each smartphone CPU to do the conversion. We calculated both the average, and the weighted average of the instruction set for each year (Table S28). We then multiplied the weighted average of the instruction set by the number of computations to calculate the number of bits of information processed each year.

The electricity consumption of smartphones was estimated using their battery capacity. We collected data about voltage and electric charge for all studied smartphones. We calculated battery capacity by multiplying voltage and electric charge (for mean, median and standard deviation values for battery capacity see Table S36). We then determined the average battery capacity of new smartphones and the weighted average capacity of the stock of smartphones (Table S29). We assumed that every smartphone was charged daily, from 0% to 100%, meaning

it was fully charged 365 times per year. To estimate smartphone electricity consumption, we multiplied the weighted average battery capacity of the stock by 365 and by the number of smartphones in use (Equation 9). A schematic representation of the methodology used to calculate electricity consumption of smartphones is shown in Figure S6.

$$\begin{gathered}Energy\ consumed = \\ = Weighted\ average\ battery\ capacity \times 365 \times Nº\ of\ Smartphones\end{gathered} \tag{9}$$

Datacentres

Datacentres are comprised of various devices, but only one is used for computing: the server. Since a datacentre has a variable number of servers, we are going to use servers as the starting point to calculate information processed and electricity consumed by datacentres. Besides servers, a datacentre uses electricity in the cooling system, storage and internal network. We will estimate the electricity consumption of each of these different components of the datacentres.

The first step to calculate the information processed was to estimate the number of servers in use. Servers are usually separated in three different categories: volume, mid-range and high-end. These categories are usually differentiated by the price. As the names suggest, volume servers have the lowest performance, i.e. lowest information processing capabilities and price while high-end servers have the highest performance and are the most expensive. We collected data for annual shipments and for the installed base of servers. Between 1981 and 1995, we had data for annual shipments, as a sum of all categories of servers, from Hilbert and López[13]. We used this data to estimate the installed base assuming a useful life of 4 years[31]. To calculate the shipments between 1975 and 1980, we extrapolated backwards, using the compound annual growth rate (CAGR) of the period 1981-1985 and the number of shipments in 1981. From 1996 until 2003 we collected data for the installed base[32–34], for the three different categories of servers. Then, we had data for the installed base for 2005 and 2010[35], again for the three categories of servers. In the years with missing data, 2004 and the period 2006-2009, we linearly interpolated between the closest years with data. After 2010, we used the installed base data from Masanet et al.[36], which had data for the three categories of servers and information on the types of datacentres where the volume servers were installed (Traditional, Cloud (non-hyperscale) and Hyperscale datacentres). After 2014, we updated Masanet et al.[36] data for north America using the latest report for the USA[37]. This required recalculating global stock values to reflect the revised U.S. figures assuming that the distribution of server types remained unchanged, meaning that if the total server count increased, each server type increased proportionally. With all these data points, we were able to create an installed base dataset from 1975 until 2022, which corresponds to step 1 in Figure S7.

The next step, step 2, was to gather information about the performance of the stock of servers. We can divide the performance results in 4 different periods. For the years 2000 and 2003 we had information from Koomey[33] that specified which specific servers, i.e. which brand and model, had a bigger share of the installed base. Koomey's data was for the three categories of servers, volume, mid-range and high-end. We then used SPEC 2000[38] values to calculate the MIPS values for each server. We calculated the logarithm of the average performance for 2000 and 2003, then linearly interpolated between these logarithmic values for 2001 and 2002.

Finally, we exponentiated the interpolated values to obtain average performances for 2001 and 2002, as the growth was exponential. Regarding the period after 2005, we started by using Koomey's [33] report which also specified which servers were the most shipped in 2005. We combined this data with the information from 2000 and 2003 and tracked the various generations of each server model (most server models include the generation in their name or follow a numbered sequence) up to 2022.

We calculated performance using the publicly available data of the benchmarks SPEC 2000 and SPEC 2006, which we then converted to MIPS. Since we did not have the number of new servers per year for each type of server, we assumed that the weighted average performance of the stock was equal to the average performance of the servers still in use in that year. We knew the release year of all servers for which we calculated performance values, and we assumed that volume servers were in use 4 years while mid-range and high-end server were in use 6 years, based on Shehabi et al.[31] and The Green Grid[39]. Weighted average performance for the year 2004 was calculated by interpolating between the values of 2003 and 2005, using the same method as for 2001 and 2002.

For the period 1996-1999, we calculated weighted average performance using the CAGR between the values of average performance in 2000 and 2003, for each category of servers. Before 1996, we only had data for the sum of all categories of servers. Thus, we calculated the aggregate weighted average performance of servers, i.e. the average performance of servers taking into account the share of the installed base of the three categories, for the years 2000 and 2005 and then calculated the CAGR between those two years. We then extrapolated backwards until 1975 using the CAGR calculated and the aggregated performance of 1996 as a base. The performance values for each type of server are shown in Table S30.

Afterwards, we calculated the number of instructions servers could process per year. To do this we used equation 3. We already explained how we obtained stock and weighted average performance of stock, so we only need to explain our assumptions about hours of use. We assumed that from 1991 (inclusive) onwards, servers would operate continuously throughout the year (8,760 hours)[36]. Prior to 1991, servers were assumed to operate 12 hours per day, totalling 4,380 hours annually. This assumption was based on the idea that, before the World Wide Web became publicly accessible, most servers operated only for part of the day.

The last step included converting instructions to bits. To do this, we used the same values that were calculated for PCs. We multiplied the weighted average of word length by the number of computations to calculate the number of bits of information processed each year.

Datacentres have various components that consume electricity, servers consuming only a portion of that energy with the remainder being used by the cooling system, storage and internal network. We will start by explaining how we calculated electricity consumed by servers, summarized in equation 10. After 2010, we had data about power consumption from the same source used for the installed base[36]. We updated the installed base, as explained earlier, while maintaining the same power draw for all types of servers, except high-end servers, which we changed to 10 kW based on the latest USA report[37]. This reference had data for the three types of servers and also for the different spaces they were located on. For the years 2000, 2003, 2004 and 2005 we retrieved data about power consumption from Koomey[33]. For the missing years, 2001, 2002, 2006, 2007, 2008 and 2009, we linearly interpolated between the closest years with data. Between 1996 and 1999, we calculated power consumption for each type of server extrapolating backwards using the CAGR calculated between the power consumption in 2000

and 2003. Before 1996, we assumed all servers were volume servers, the category with the biggest number of servers in 1996, and used the 2000 to 2003 CAGR of this type of servers to extrapolate backwards until 1975. These values of power consumption are power draw values, meaning they indicate the operational power per server. All power draw values are shown in Table S31. To calculate electricity consumption of a specific year, we multiply power draw by the number of servers in use and the hours of use, both of which we have already addressed before. We did this calculation for the three categories of servers, when category specific data for power and number of servers was available. A schematic representation of the method used to calculate electricity consumption is shown in Figure S8.

$$\begin{gathered} Energy\ consumed = \\ = Average\ power\ draw \times N^{o}\ of\ Servers\ \times Hours\ of\ use \end{gathered} \tag{10}$$

We applied the same method to estimate the electricity consumption of both storage and the internal network. After 2010, we used Masanet et al.[36] data for electricity consumption in networks and storage. After 2014, these values were updated to take into account the most recent USA data[37]. For the years 2000 and 2005, we retrieved data for the electricity consumption of networks and storage from Koomey[34] and calculated their share relative to servers' electricity consumption. For 2010, using data from Masanet et al.[36], we were able to calculate the electricity consumption of networks and storage as a share of the server electricity consumption. For the remaining years between 2000 and 2010, we just linearly interpolated between the shares calculated for those 3 years. For the years between 1996 and 1999, we extrapolated backward from 2000 using the compound annual growth rate (CAGR) calculated between the 2000 and 2005 shares. After obtaining the shares for both networks and storage, for each year in the 1996-2009 period, we multiplied them by the electricity consumption of servers that we had already calculated, which allowed us to determine the electricity consumed by storage and networks. Prior to 1996 we assumed that networks and storage consumed the same share they consumed in 1996, meaning that they had a constant share of the servers' electricity consumption from 1975 until 1996.

The last component of the datacentres that consumes electricity is the infrastructure, mainly cooling. To calculate this value, we used power usage effectiveness (PUE), a metric that is widely used to characterize datacentre energy efficiency. This metric is the ratio between total electricity demand of a datacentre and electricity used by information technology (IT), equation 11; in other words, if the PUE is 2 non-IT uses as much electricity as IT (IT includes servers, storage and networks). A PUE of 1 would mean that no electricity is used for cooling and other infrastructure uses. To calculate electricity consumption of infrastructure after 2010, we used Masanet et al.[36] data for PUE along with the data for IT electricity consumption, which we already had. Before 2010, we used the assumption made by Koomey[34] that PUE was equal to 2. This assumption is likely to result in an underestimation of the electricity consumed by datacentres before 2010. However, since no data on the world average PUE was available, the assumption was necessary to estimate datacentre electricity consumption. Finally, datacentre electricity consumption was calculated as the sum of the electricity consumption of its 4 different components, servers, storage, network and infrastructure. Electricity consumption of the 4 different components is shown in Table S32.

$$PUE = \frac{Total\ datacenter\ electricity\ consumption}{IT\ equipment\ electricity\ consumption} \tag{11}$$

References

1. Nordhaus, W.D. (2007). Two centuries of productivity growth in computing. Journal of Economic History *67*, 128–159. https://doi.org/10.1017/S0022050707000058.
2. Koomey, J.G., Berard, S., Sanchez, M., and Wong, H. (2011). Implications of historical trends in the electrical efficiency of computing. IEEE Annals of the History of Computing *33*, 46–54. https://doi.org/10.1109/MAHC.2010.28.
3. Norford, L., Hatcher, A., Harris, J., Roturier, J., and Yu, O. (1990). Electricity use in Information Technologies. Annual Review of Energy *15*, 423–453. https://doi.org/10.1146/annurev.eg.15.110190.002231.
4. Roth Fred Goldstein Jonathan Kleinman Arthur D, K.W., and Little, A.D. (2002). Energy Consumption by Office and Telecommunications Equipment in Commercial Buildings Volume I: Energy Consumption Baseline.
5. Dongarra, J., Sterling, T., Simon, H., and Strohmaier, E. (2005). High-Performance Computing: Clusters, Constellations, MPPs, and Future Directions. Comput Sci Eng *7*, 51–59. https://doi.org/10.1109/MCSE.2005.34.
6. Dongarra, J., Foster, I., Fox, G., Gropp, W., Kennedy, K., Torczon, L., and White, A. (2003). Sourcebook of parallel computing (Morgan Kaufmann Publishers) https://doi.org/10.5555/941480.
7. Top500 (2025). TOP500. https://www.top500.org/.
8. Dongarra, J.J. (1988). The LINPACK Benchmark: An explanation. In, pp. 456–474. https://doi.org/10.1007/3-540-18991-2_27.
9. NCAR (2023). NCAR supercomputing history. https://www2.cisl.ucar.edu/ncar-supercomputing-history.
10. Giladi, R. (1996). Evaluating the Mflops measure. IEEE Micro *16*, 69–75. https://doi.org/10.1109/40.526927.
11. Prieto, A., Prieto, B., Escobar, J.J., and Lampert, T. (2025). Evolution of computing energy efficiency: Koomey's law revisited. Cluster Comput *28*, 42. https://doi.org/10.1007/s10586-024-04767-y.
12. Statista (2023). Personal computer (PC) shipments worldwide from 2006 to 2023. Gartner. https://www.statista.com/statistics/273495/global-shipments-of-personal-computers-since-2006/.
13. Hilbert, M., and López, P. (2011). The World's Technological Capacity to Store, Communicate, and Compute Information. Science (1979) *332*, 60–65. https://doi.org/10.1126/science.1200970.
14. Reimer, J. (2012). Total Share: Personal Computer Market Share 1975-2010. https://jeremyreimer.com/rockets-item.lsp?p=137.
15. Statista (2023). Desktop PCs global shipments 2010-2026. IDC. https://www.statista.com/statistics/269044/worldwide-desktop-pc-shipments-forecast/.

16. Statista (2023). Notebook PC global shipments 2010-2026. IDC. https://www.statista.com/statistics/269048/worldwide-portable-pc-shipment-forecast/.

17. Statista (2023). Notebook unit shipments worldwide from 2005 to 2023. TrendForce. http://www.statista.com/statistics/203691/global-unit-shipments-of-notebooks/.

18. Daily News of Los Angeles (CA) (1987). LAP-TOP COMPUTERS GAIN STATURE AS POWER GROWS.

19. Statista (2023). Number of computer households worldwide 2013-2028. Statista. https://www.statista.com/forecasts/1146269/computer-households-in-the-world.

20. Statista (2023). Computer penetration rate among households worldwide 2005-2019. ITU. https://www.statista.com/statistics/748551/worldwide-households-with-computer/.

21. Blundell, G. (1983). Personal computer in the eighties. Byte magazine, 166–182.

22. Primate Labs Inc (2024). Geekbench 6. https://www.geekbench.com/.

23. Datareportal (2023). Global Overview Report - 2019 - 2022. https://datareportal.com/reports/?tag=Global+Overview.

24. Liang, Y., Zhao, C.Z., Yuan, H., Chen, Y., Zhang, W., Huang, J.Q., Yu, D., Liu, Y., Titirici, M.M., Chueh, Y.L., et al. (2019). A review of rechargeable batteries for portable electronic devices. InfoMat *1*, 6–32. https://doi.org/10.1002/INF2.12000.

25. Harris, J., Roturiertt, J., Norford, L.K., and Rabl, A. (1988). Technology assessment: electronic office equipment https://doi.org/10.13140/RG.2.1.2877.6403.

26. Ericsson (2023). Ericsson Mobility Visualizer. https://www.ericsson.com/en/reports-and-papers/mobility-report/mobility-visualizer?f=1&ft=3&r=1&t=8&s=1&u=1&y=2011,2022&c=1.

27. Statista (2023). Global smartphone sales to end users 2007-2022. Gartner. https://www.statista.com/statistics/263437/global-smartphone-sales-to-end-users-since-2007/.

28. Sima, D. (2018). ARM's processor lines. https://users.nik.uni-obuda.hu/sima/letoltes/Processor_families_Knowledge_Base_2019/ARM_processors_lecture_2018_12_02.pdf.

29. Carroll, A., and Heiser, G. (2013). The systems hacker's guide to the galaxy energy usage in a modern smartphone. In Proceedings of the 4th Asia-Pacific Workshop on Systems (ACM), pp. 1–7. https://doi.org/10.1145/2500727.2500734.

30. Carroll, A., and Heiser, G. (2010). An analysis of power consumption in a smartphone. Proceedings of the 2010 USENIX conference on USENIX annual technical conference.

31. Shehabi, A., Smith, S., Horner, N., Azevedo, I., Brown, R., Koomey, J., Masanet, E., Sartor, D., Herrlin, M., and Lintner, W. (2016). United States Data Center Energy Usage Report.

32. Koomey, J.G. (2007). Estimating regional power consumption by servers: A technical note.

33. Koomey, J.G. (2007). ESTIMATING TOTAL POWER CONSUMPTION BY SERVERS IN THE U.S. AND THE WORLD.

34. Koomey, J.G. (2008). Worldwide electricity used in data centers. Environmental Research Letters *3*. https://doi.org/10.1088/1748-9326/3/3/034008.

35. Koomey, J.G. (2011). GROWTH IN DATA CENTER ELECTRICITY USE 2005 TO 2010.

36. Masanet, E., Shehabi, A., Lei, N., Smith, S., and Koomey, J. (2020). Recalibrating global data center energy-use estimates. Science (1979) *367*, 984–986. https://doi.org/10.1126/science.aba3758.

37. Shehabi, A., Smith, S.J., Hubbard, A., Newkirk, A., Lei, N., Abu Bakar Siddik, M., Holecek, B., Koomey, J., Masanet, E., and Sartor, D. (2024). 2024 United States Data Center Energy Usage Report. Lawrence Berkeley National Laboratory. https://doi.org/https://doi.org/10.71468/P1WC7Q.

38. SPEC (2007). SPEC 2000. https://www.spec.org/cpu2000/.

39. Aggar, M., Banks, M., Dietrich, J., Shatten, B., Stutz, M., and Tong-Viet, D.E. (2012). Data centre life cycle assessment guidelines.

40. Malmodin, J., and Lundén, D. (2018). The energy and carbon footprint of the global ICT and E & M sectors 2010-2015. Sustainability (Switzerland) *10*. https://doi.org/10.3390/su10093027.

41. Malmodin, J., Moberg, A.S., Lundén, D., Finnveden, G., and Lövehagen, N. (2010). Greenhouse gas emissions and operational electricity use in the ICT and entertainment & Media sectors. J Ind Ecol *14*, 770–790. https://doi.org/10.1111/j.1530-9290.2010.00278.x.

42. Malmodin, J., Lövehagen, N., Bergmark, P., and Lundén, D. (2024). ICT sector electricity consumption and greenhouse gas emissions – 2020 outcome. Telecomm Policy *48*. https://doi.org/10.1016/j.telpol.2023.102701.

43. Andrae, A. (2020). New perspectives on internet electricity use in 2030. Engineering and Applied Science Letters (EASL) *3*, 19–31. https://doi.org/10.30538/psrp-easl2020.0038.

44. Teehan, P., and Kandlikar, M. (2012). Sources of Variation in Life Cycle Assessments of Desktop Computers. J Ind Ecol *16*. https://doi.org/10.1111/j.1530-9290.2011.00431.x.
